# Growth of *Limnospira platensis* Under Elevated CO_2_ with the Addition of Sodium Selenite

**DOI:** 10.3390/plants15162472

**Published:** 2026-08-14

**Authors:** Anatoly V. Grigorenko, Elizaveta M. Kovalenko, Marina E. Vavilkina, Maksim A. Kravets, Mikhail S. Vlaskin

**Affiliations:** Joint Institute for High Temperatures of the Russian Academy of Sciences, 125412 Moscow, Russia; presley1@mail.ru (A.V.G.); lizloom@mail.ru (E.M.K.); vavilkina-marina@rambler.ru (M.E.V.); kravets.ma@phystech.edu (M.A.K.)

**Keywords:** *Limnospira platensis*, sodium selenite, selenium bioaccumulation, elevated CO_2_, growth rate, toxicity threshold, nutrient consumption, biomass viability, photobioreactor, cyanobacteria, adaptation

## Abstract

Cultivation of *L. platensis* under elevated CO_2_ ensures CO_2_ biofixation, while adding Na_2_SeO_3_ to the culture medium, produces valuable Se-enriched biomass. This study evaluated the effect of Na_2_SeO_3_ supplementation (20–640 mg/L) on the growth of *L. platensis* under 3 vol.% CO_2_. The cultivation was carried out in 10 L column-type photobioreactors under continuous (24 h·d^−1^) illumination with intensity of 220 µmol·m^−2^·s^−1^, a constant temperature of 27 °C and aeration rate of 1 L/min. Two consecutive cultivation cycles (8 days each) were conducted: the first with the original strain and the second with an inoculum adapted to 20 mg/L (taken from the first cycle). The growth rate, cell viability, pH, and the concentrations of nitrates, phosphates, carbonates, bicarbonates, NH_4_^+^ ion were investigated. It was established that under intensive cultivation at elevated CO_2_, *L. platensis* is very sensitive to Na_2_SeO_3_ supplements: concentrations > 160 mg/L led to culture death within the first 2–4 days, while the acute toxicity threshold lies in the range of 80–100 mg/L. The highest biomass growth rate in the 1st and 2nd cycles was achieved in the control (0 mg/L of Na_2_SeO_3_): 329 and 239 mg·L^−1^·d^−1^ dry weight, respectively. At 20 mg/L, the growth rate drops to 289 and 208 mg·L^−1^·d^−1^ in the 1st and 2nd cycles, respectively. The percentage of live trichomes at the end of the 1st and 2nd cycles at 20 mg/L was 85 and 67%, respectively, indicating low adaptation capacity even to this concentration of Na_2_SeO_3_. The addition of Na_2_SeO_3_ did not lead to significant pH deviations from the control. Nitrates and phosphates consumption slowed down at Na_2_SeO_3_ concentrations of 40–60 mg/L. Bicarbonate and carbonate ions did not change significantly across all samples due to the maintenance of elevated CO_2_ concentration. In a separate experiment under atmospheric CO_2_ (0.04%), selenite toxicity was less pronounced than under 3% CO_2_, and growth declined after day 4 due to carbon limitation rather than selenite toxicity.

## 1. Introduction

*Limnospira platensis* (Gomont) K.R.S.Santos & Hentschke, commonly known as spirulina, is a cyanobacterium that consists of free-floating, multicellular trichomes. In nature, *L. platensis* is found in tropical and subtropical lakes with a water pH of 8–11 and high concentrations of carbonates and bicarbonates [1,2].

Today, the biomass of *Limnospira platensis* is widely used in the food and feed industries [3,4]. The nutritional value of *L. platensis* is due to the rich chemical composition of its biomass. Dry *L. platensis* biomass contains approximately 60–70% protein, including all essential amino acids, making it a complete protein source [5]. Lipids account for about 5–10% of the dry biomass, including many unsaturated fatty acids, such as gamma-linolenic acid. Furthermore, *L. platensis* contains various B-group vitamins, vitamin E, and vitamin K. It is also rich in pigments with antioxidant properties [6].

*Limnospira platensis* is characterized by a rich mineral composition, including essential macronutrients (K, Na, Ca, Mg, P) [7]. It also possesses a pronounced ability to accumulate mineral elements. The mineral composition of *L. platensis* depends on the composition of the nutrient medium, which allows for the purposeful enrichment of the biomass with deficient trace elements [8].

One such deficient trace element of interest is selenium. Interest in selenium stems from its role as a powerful antioxidant that protects cells from aging and damage [9,10]. It was reported in [11] that selenium-enriched spirulina has high potential for alleviating osteoporosis by suppressing inflammatory processes. Selenium deficiency can cause weakened immunity, thyroid dysfunction, and the progression of neurodegenerative diseases [12,13]. It is estimated that selenium deficiency affects 15% of the population and is particularly characteristic of regions with selenium-deficient soils (China, Europe, New Zealand) [14]. The advantage of selenium-enriched spirulina is its ability to transform inorganic selenium into bioavailable forms by binding it with proteins, lipids, and polysaccharides [15,16].

Several studies have already attempted to produce selenium-enriched *Limnospira platensis* biomass. These works primarily focused on studying the tolerance of *L. platensis* to elevated selenium concentrations in the nutrient medium. It was shown in [15] that sodium selenite concentrations below 400 mg/L stimulate the growth of *L. platensis*, particularly in the range of 0.5 to 40 mg/L, while concentrations above 500 mg/L exert a toxic effect. Another study [17] demonstrated that sodium selenite supplements at concentrations of 300–700 µmol/L (23–55 mg/L) had a positive effect on the growth of *L. platensis*. A concentration of 880 µmol/L (70 mg/L) had a negligible effect. In [18], it was confirmed that *L. platensis* easily tolerates sodium selenite concentrations of 100–200 mg/L, with biomass accumulation remaining within the range of the control group. Lower concentrations of sodium selenite have a stimulating effect on biomass accumulation. Work [19] examined the effect of batch selenium additions on the growth of *L. platensis* and the accumulation of organic selenium, showing that an addition of 100 mg/L moderately stimulates the growth of *L. platensis*.

In addition to its high nutritional value, *Limnospira platensis* possesses a high capacity for CO_2_ utilization in certain applications. Recent studies have demonstrated its potential for CO_2_ capture from industrial sources [20] and from atmospheric CO_2_ under controlled conditions [21]. Although large-scale atmospheric CO_2_ mitigation remains challenging, these findings suggest that *L. platensis*-based systems may contribute to decentralized carbon capture and utilization strategies, particularly when coupled with industrial emission sources. Regarding the integration of *L. platensis* cultivation systems with concentrated CO_2_ sources, study [22] investigated the cultivation process under elevated atmospheric CO_2_ concentrations. It was shown that *L. platensis* demonstrates good biomass quality and a high growth rate at CO_2_ concentrations of 1 and 5 vol.%. Another study [20] explored the use of exhaust gases by *L. platensis* cultures, showing that the use of exhaust gases led to a more effective increase in biomass without changing its quality. Overall, the above-mentioned studies demonstrated sufficiently high biomass growth rates and CO_2_ absorption efficiency when cultivated in a gas-air environment with elevated CO_2_ concentrations. Based on these previous findings, we selected 3% CO_2_ as an intermediate concentration that is representative of industrial flue gas CO_2_ levels (typically 3–15%) and has been shown to support high growth rates in *L. platensis* without causing acidification of the medium. Additionally, our preliminary experiments (Appendix B) confirmed that at 3% CO_2_ the pH of the culture medium remains stable within the optimal range for spirulina growth (8.6–9.0) for at least two days, indicating that the system reaches equilibrium with the CO_2_-enriched atmosphere without excessive pH fluctuations.

While the effect of selenium on *Limnospira platensis* has been studied previously, most of these studies were conducted under ambient CO_2_ conditions and focused on selenium accumulation rather than on the physiological response to combined stress. The synergistic effect of elevated CO_2_ partial pressure and acute selenite toxicity remains poorly understood. High CO_2_ concentrations not only provide additional carbon for photosynthesis but also substantially alter the pH of the culture medium, which in turn affects selenium speciation, bioavailability, and toxicity. These pH-dependent changes may fundamentally modify the detoxification mechanisms and overall tolerance of *Limnospira* to selenite. However, systematic data on growth performance, viability, and medium chemistry under such combined conditions are still lacking.

The novelty of our study is that we provide the first systematic data on how elevated CO_2_ (3%) affects the tolerance of *Limnospira platensis* to a wide range of selenite concentrations. This is important not only for understanding the basic physiology of this cyanobacterium, but also for practical applications such as using flue gas CO_2_ for selenium-enriched biomass production.

Therefore, the aim of this work is to investigate the growth process *of Limnospira platensis* in a nutrient medium supplemented with sodium selenite under elevated CO_2_ concentration. The objectives of the work are:

1. To determine the effect of sodium selenite concentration on the viability and growth rate of *Limnospira platensis*.
2. To determine the effect of sodium selenite concentration on the pH of the nutrient medium during growth.
3. To determine the effect of sodium selenite concentration on nitrate and phosphate content in the nutrient medium during growth.
4. To determine the effect of sodium selenite concentration on carbonate and bicarbonate content in the nutrient medium during growth.
5. To determine the effect of sodium selenite concentration on content of NH**_4_^+^** ions in the nutrient medium during growth.
6. To compare the effects of sodium selenite on *Limnospira platensis* under elevated (3%) and atmospheric CO**_2_** conditions.

## 2. Results

### 2.1. Biomass Yield

On the 8th day of Experiment 1, following the collection of samples for analysis, the contents of the photobioreactors were drained, filtered, and dried for further evaluation. The final mass of the dried biomass per photobioreactor and the dry biomass concentration within the suspension are presented in Table 1, which includes data only for selenite concentrations up to 80 mg/L, as at higher concentrations the culture died before day 8 and no biomass was available for analysis.

The final conversion factor for translating optical density into dry cyanobacterial concentration, derived from the total mass of the dried biomass, is:

$$\frac{C_{spirulina}[g/L]}{{OD}_{655}}=(0.90\pm 0.05)g/L$$

Within the margin of error, the calculated conversion factor aligns with the empirical coefficient $\frac{C_{spirulina}[g/L]}{{OD}_{655}}$ = 0.85 used in this study, which was established in previous experimental research. The error is caused by losses during biomass harvesting and drying.

### 2.2. Viability of Spirulina

Results from the first experiment revealed a clear correlation between sodium selenite concentration and the viability of spirulina adapted to a high CO_2_ concentration (3 vol%). High concentrations of sodium selenite led to a loss of culture viability within a short period. Viability remained relatively stable up to 20 mg/L, but declined progressively at 40–80 mg/L and dropped sharply at 160 mg/L and above.

**Figure 1:**
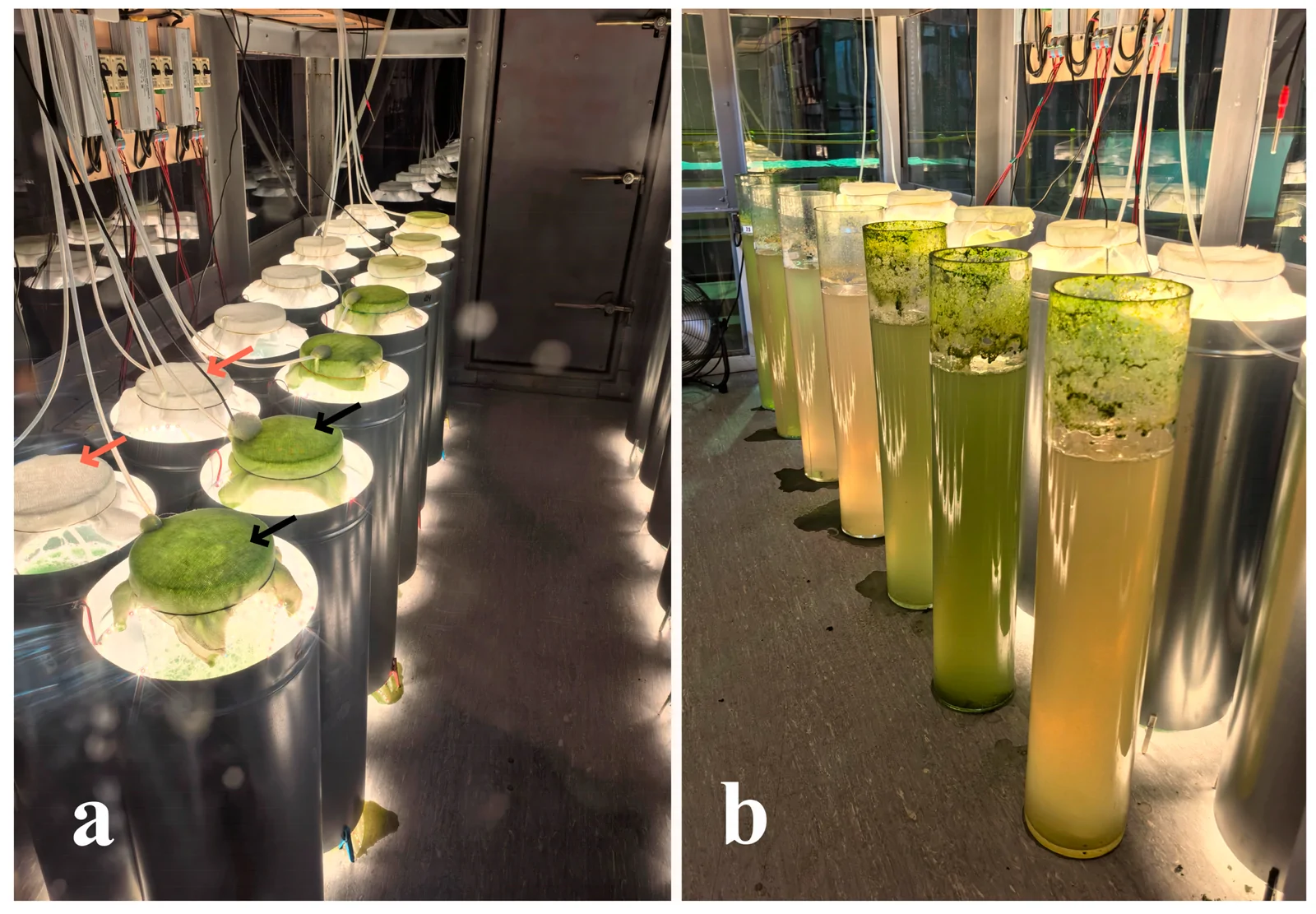
Photographs of PBRs during the first experiment. (**a**) Red arrows (**left**) indicate PBRs with living spirulina a few hours after the start (0 and 20 mg/L sodium selenite); black arrows (**right**) indicate PBRs with dead spirulina (320 and 640 mg/L). (**b**) Bioreactors with dead spirulina and discolored contents on the 2nd day.

Bioreactors containing living spirulina maintained a vibrant green color and clean gauze lids. In contrast, during the first experiment, bioreactors with dead spirulina exhibited discolored (bleached) contents; cell lysis occurred, leading to contamination of the PBR lids and leakage of the contents (Figure 1).

In the second experiment, aimed at identifying the biomass death threshold, the threshold was found to be within the 80–100 mg/L range. All samples cultivated at sodium selenite concentrations of 100, 120, 140, and 160 mg/L showed a loss of viability by the 6th day. A borderline effect was observed at 80 mg/L: in two out of three PBRs, the culture remained viable, although growth was inhibited. This suggests that 80 mg/L is at the limit of tolerance for the specific *Limnospira platensis* strain and cultivation conditions used. Growth heterogeneity was also observed at 40 and 60 mg/L. At 40 mg/L, only one of the three PBRs showed good growth; at 60 mg/L, two out of three cultures remained viable. The culture continuing growth at 20 mg/L confirmed its adaptation but demonstrated a growth slowdown between days 6 and 8.

The data suggest that using an inoculum grown with 20 mg/L sodium selenite shifted the acute toxicity threshold. In the first experiment, the culture died at 160 mg/L by day 4, whereas in the second experiment, it survived at the same concentration until day 6. This effect may be linked to the activation of protective mechanisms, such as increased antioxidant production or the conversion of selenium into less toxic compounds. However, 80 mg/L remains the critical point for viability. The high variability at 40–80 mg/L can be attributed to physiological heterogeneity within the population or the unpredictable accumulation of cellular damage.

**Figure 2:**
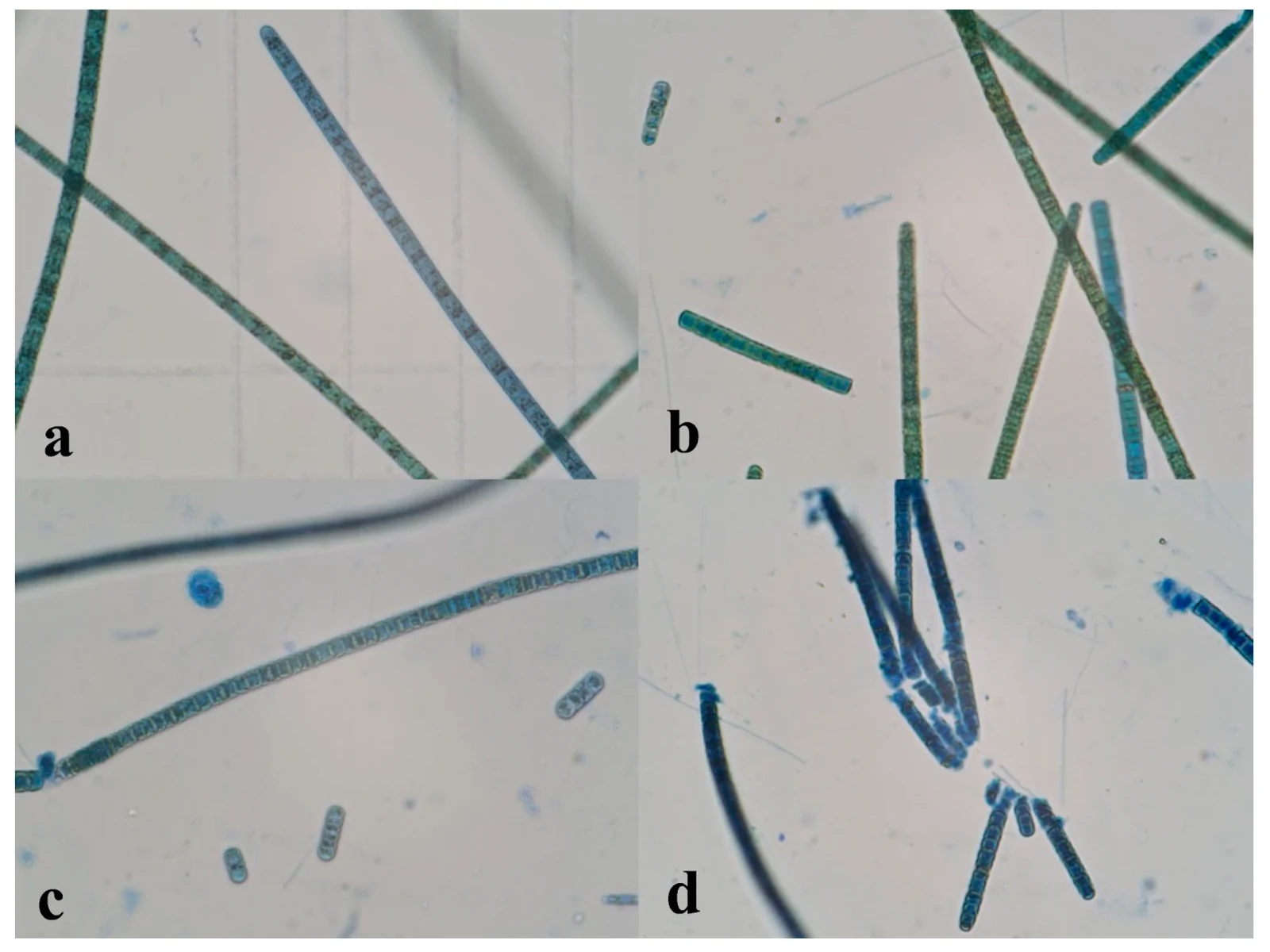
Microscopic evaluation of the culture on day 8 of experiment No. 2. (**a**) control sample (live green trichomes and dead ones stained with methylene blue); (**b**) 20 mg/L sodium selenite; (**c**) 40 mg/L sodium selenite; (**d**) 60 mg/L sodium selenite.

In addition to visual assessments, microscopic evaluations were performed (Figure 2). In the control sample, morphology was normal: long, intact trichomes predominated. In selenium-treated samples, several changes were noted: an increase in non-viable trichomes (Figure 2b), fragmentation from long filaments to short chains or single cells (Figure 2c), and a loss of cellular integrity (Figure 2d). These findings confirm the cytotoxic effect of sodium selenite on *Limnospira platensis*.

**Figure 3:**
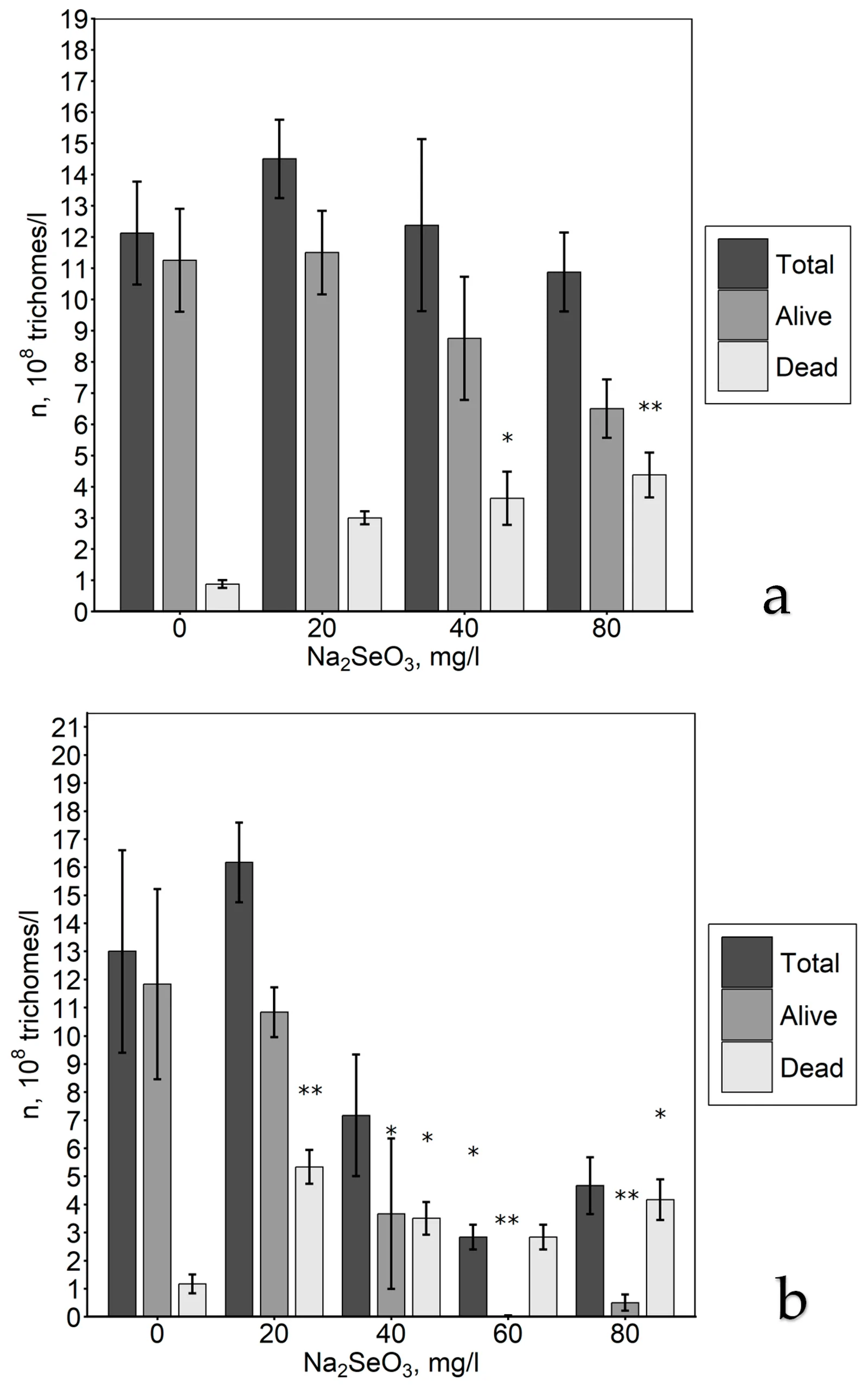
Dependence of the total number of trichomes per liter, as well as live and dead trichomes, on the sodium selenite concentration in the nutrient medium at the end of the first (**a**) and second (**b**) experiments (Day 8). Error bars represent 95% confidence intervals (Student’s t-distribution). * *p* < 0.05, ** *p* < 0.01 (Dunnett’s test vs. control).

**Figure 4:**
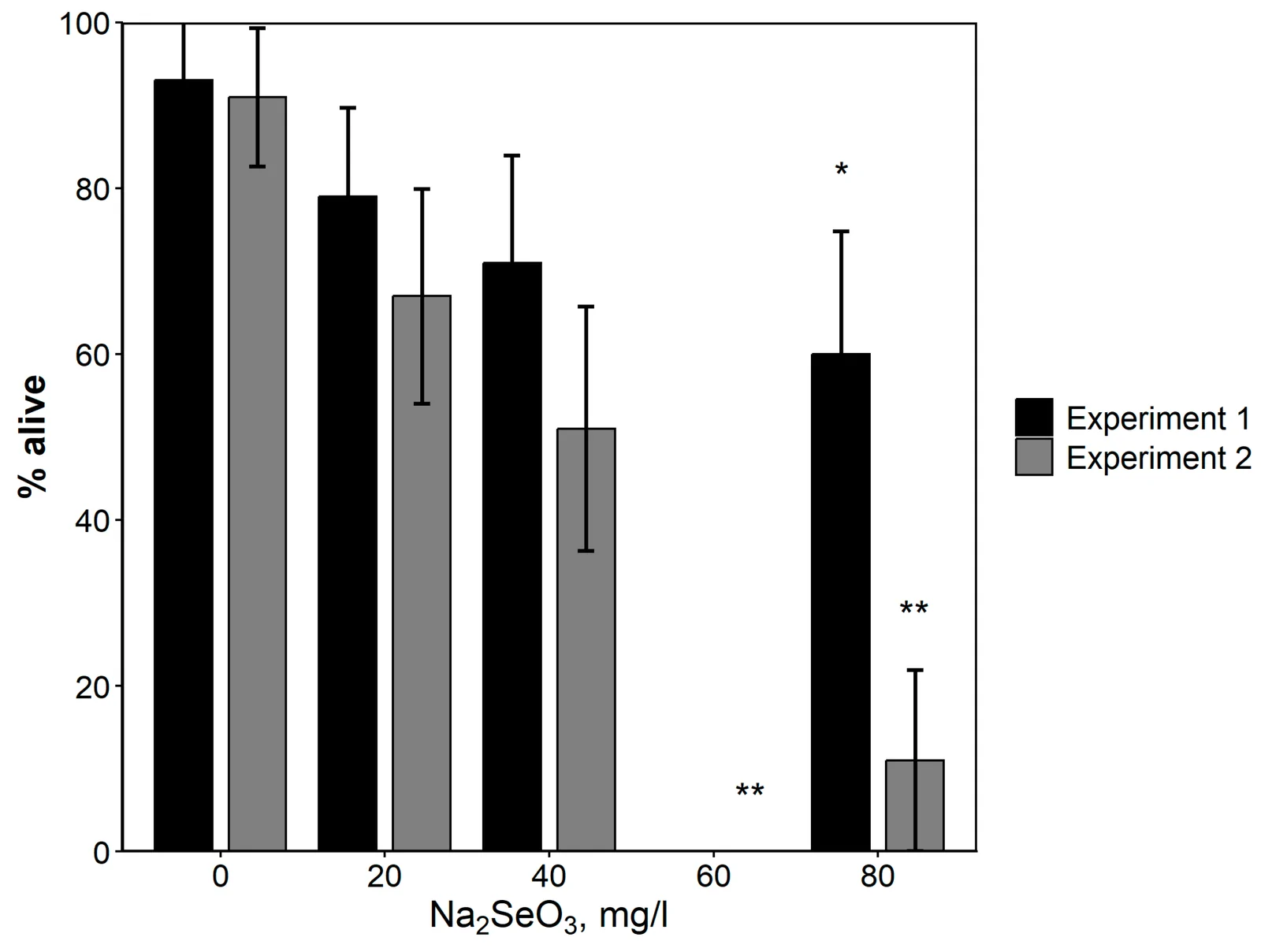
Percentage of live *Limnospira platensis* trichomes depending on sodium selenite concentration in the first and second experiments (Day 8). Error bars represent SE of mean. * *p* < 0.05, ** *p* < 0.01 (Dunnett’s test vs. control).

To quantitatively assess the culture’s viability in the first experiment, a diagram was constructed (Figure 3a) showing the relationship between the total concentration of individuals, the concentration of live and dead trichomes, and the sodium selenite concentration at the end of the experiment. The dependence of viability on sodium selenite concentration on the 8th day of cultivation is shown in Figure 4. The diagram indicates that at a concentration of 0 mg/L (control), the total number of trichomes is 12 × 10^8^ L^−1^, with a high viability percentage of 93%. As the sodium selenite concentration increases to 40 mg/L, a slight increase in the total number of trichomes is observed; however, the viability percentage drops to 71%. A further increase in concentration to 80 mg/L leads to a stabilization of the total number of individuals at 12 × 10^8^ L^−1^, with viability decreasing to 60%.

Thus, in the first experiment, as the selenium concentration increases, there is a trend toward decreased viability and a gradual reduction in total trichome density after the peak at 20 mg/L. This indicates the accumulation of toxic effects and an increasing proportion of dead cells.

In the second experiment, a similar diagram (Figure 3b) demonstrates a more pronounced dependence of these parameters on the sodium selenite concentration by the 8th day. The viability dependence on sodium selenite concentration is shown in Figure 4.

As shown in Figure 4, in the control (0 mg/L), the total number of trichomes is 13 × 10^8^ L^−1^ with a viability of 91%. At 20 mg/L, viability decreases to 67%. Subsequently, at 40 mg/L, the density drops sharply to 7 × 10^8^ L^−1^ with a viability of 51%. At 60 mg/L, the total trichomes count is only 3 × 10^8^ L^−1^ with negligible viability. Finally, at 80 mg/L, the density rises slightly to 5 × 10^8^ L^−1^, but viability remains low at 11%.

In both experiments, the threshold for a significant reduction in live trichomes was observed within the 60–80 mg/L range, although in the first experiment the decline was more gradual. This confirms that 8 days pre-adaptation to 20 mg/L sodium selenite does not significantly shift this threshold.

Detailed statistical analysis is provided in Appendix A.1. In Experiment 1, total trichomes, live trichomes, and dead trichomes were significantly affected, with a significant increase in dead trichomes observed at 80 mg/L. In Experiment 2, total trichomes and live trichomes were highly significantly affected, with a significant reduction in total trichomes at 60 mg/L and in live trichomes at both 60 mg/L and 80 mg/L. These results confirm a concentration-dependent toxic effect of selenite, with a threshold for significant inhibition observed at 60–80 mg/L.

The biomass grown at a concentration of 20 mg/L in the first experiment served as the inoculum for the second experiment. The percentage of live trichomes at the end of the first experiment at a sodium selenite concentration of 20 mg/L was 85%. However, the viability of *Limnospira platensis* on the 8th day of the second experiment with the addition of 20 mg/L sodium selenite decreased to 67%. This may indicate a low capacity of this particular *Limnospira platensis* strain to adapt to even this selenium concentration under the experimental conditions used. However, it should be noted that the duration of each experiment was only 8 days, which may not be sufficient for natural selection and genetic adaptation to occur. Longer-term cultivation studies would be required to assess whether this strain can acquire increased selenium tolerance over time.

Detailed statistical analysis is provided in Appendix A.1. Selenite exposure reduced the proportion of live trichomes in a concentration-dependent manner. In Experiment 1, the percentage of live trichomes was significantly reduced at 80 mg/L. In Experiment 2, a significant decrease was observed at 60 mg/L and 80 mg/L. Overall, the threshold for a statistically significant reduction in live trichomes was 60–80 mg/L.

Since the threshold for a significant reduction in viability remained between 60 and 80 mg/L in both experiments, no definitive change in the acute sensitivity threshold was observed.

### 2.3. Growth of Spirulina Biomass

**Figure 5:**
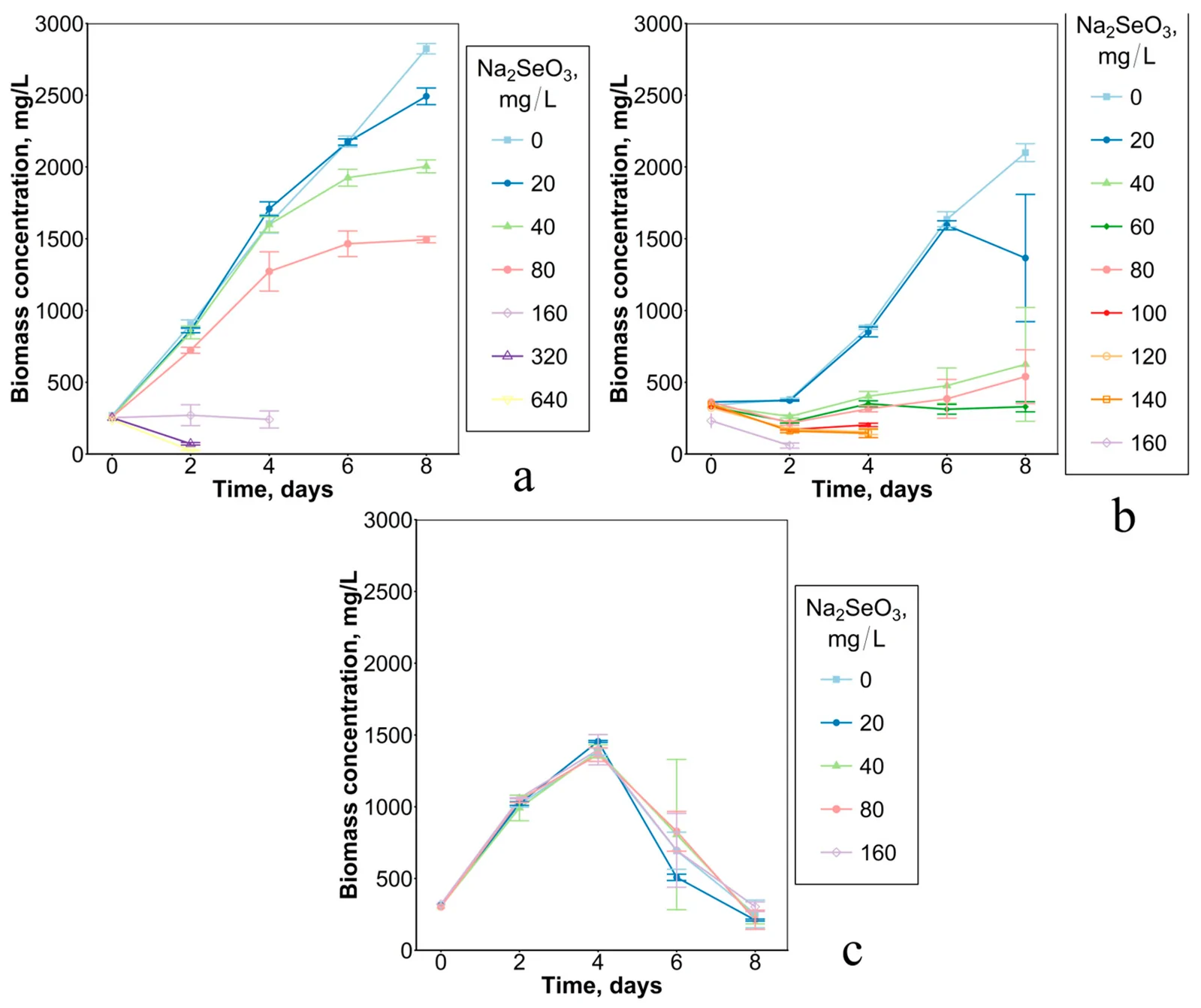
Biomass concentration of *Limnospira platensis* in suspension grown at different sodium selenite concentrations (Concentration in the legend) during: (**a**) the first experiment, (**b**) the second experiment, (**c**) the third experiment (atmospheric CO_2_). Error bars represent SE of mean.

Figure 5a presents the relationship between biomass concentration and time (days 0, 2, 4, 6, 8) for various sodium selenite concentrations (0, 20, 40, 80, 160, 320, and 640 mg/L), adjusted by the conversion factor (see Section 2.1). The graph demonstrates linear biomass growth for low selenite concentrations (0–40 mg/L), reaching maximum values on day 8. A statistically significant decrease in biomass was already observed at 20 mg/L, with more pronounced inhibition at concentrations of 160 mg/L and above.

Sodium selenite at 20 mg/L reduced biomass accumulation compared to the control (0 mg/L). Concentrations exceeding 80 mg/L induce a toxic effect, leading to stagnation or a decrease in biomass concentration.

In the second experiment, spirulina biomass previously grown and adapted in the presence of 20 mg/L sodium selenite from the first experiment was used as the inoculum. The most intensive and steady growth was observed in the control sample. Growth slows down even at a concentration of 20 mg/L. At 40 mg/L, growth is further restricted, reaching a maximum of only 662 mg/L on day 8. At 60 mg/L, the concentration peaks on day 4 and then stabilizes by day 8. For concentrations of 100 mg/L and above, rapid inhibition and culture death occur.

In the second experiment, the control (0 mg/L) showed the highest biomass accumulation among all variants within that experiment, reaching a concentration above 2000 mg/L on day 8. With further increases in sodium selenite concentration (≥40 mg/L), growth slowed down rapidly, and at concentrations ≥ 60 mg/L, mass mortality of the culture occurred. This confirms that pre-adaptation to 20 mg/L did not substantially expand the upper threshold of tolerance.

In some samples with high sodium selenite concentrations, a sharp, rapid surge in growth or sudden culture death was observed on day 8 in only one of the three replicates, which requires further investigation.

In the third experiment (Figure 5c) all variants, including the control, showed a similar pattern: biomass increased steadily until day 4, reaching values of 1600–1700 mg/L, and then declined sharply by day 8 to 250–350 mg/L. This indicates that the growth arrest on day 4 was not caused by selenite toxicity (since even the control exhibited the same drop), but rather by the depletion of bicarbonate (HCO_3_^−^) in the medium, which is the primary inorganic carbon source for *L. platensis* photosynthesis. Thus, over the tested concentration range, selenite did not significantly inhibit growth; the limiting factor was the exhaustion of available HCO_3_^−^.

Detailed statistical analysis is provided in Appendix A.2. Statistical analysis of biomass data confirmed that sodium selenite exerts a pronounced concentration-dependent and time-dependent inhibitory effect on spirulina biomass accumulation in both experiments. In Experiment 1, the threshold for statistically significant growth inhibition progressively decreased from 640 mg/L on day 0 to 20 mg/L on day 8. In Experiment 2, the threshold decreased from 160 mg/L on day 0 to 40 mg/L from day 2 onward and remained stable. The overall toxic threshold for biomass accumulation was approximately 80 mg/L.

### 2.4. Biomass Growth Rate

**Figure 6:**
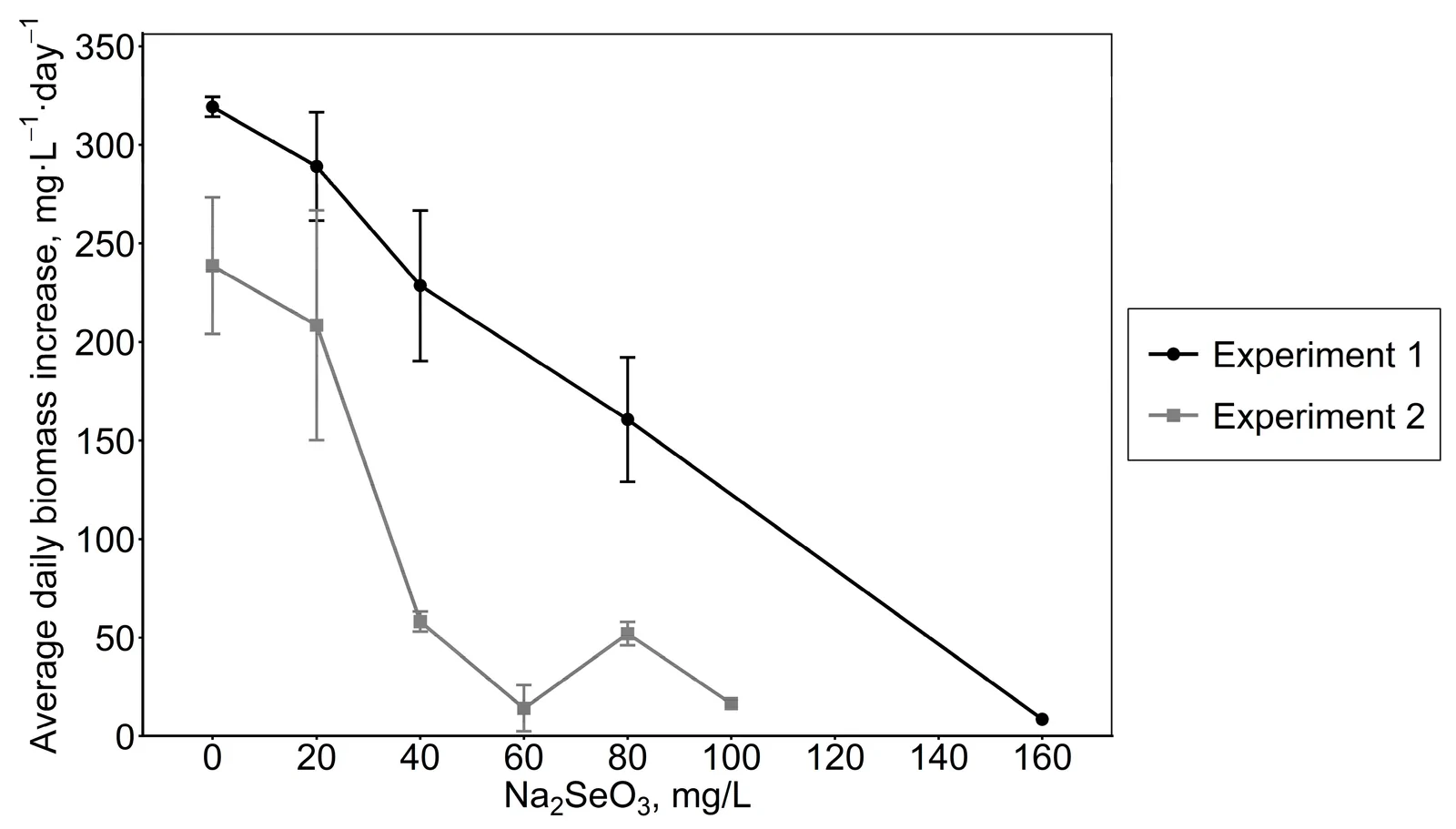
Average daily biomass productivity rate at different sodium selenite concentrations in Experiment 1 and 2. Error bars represent SE of OLS regression.

**Figure A1:**
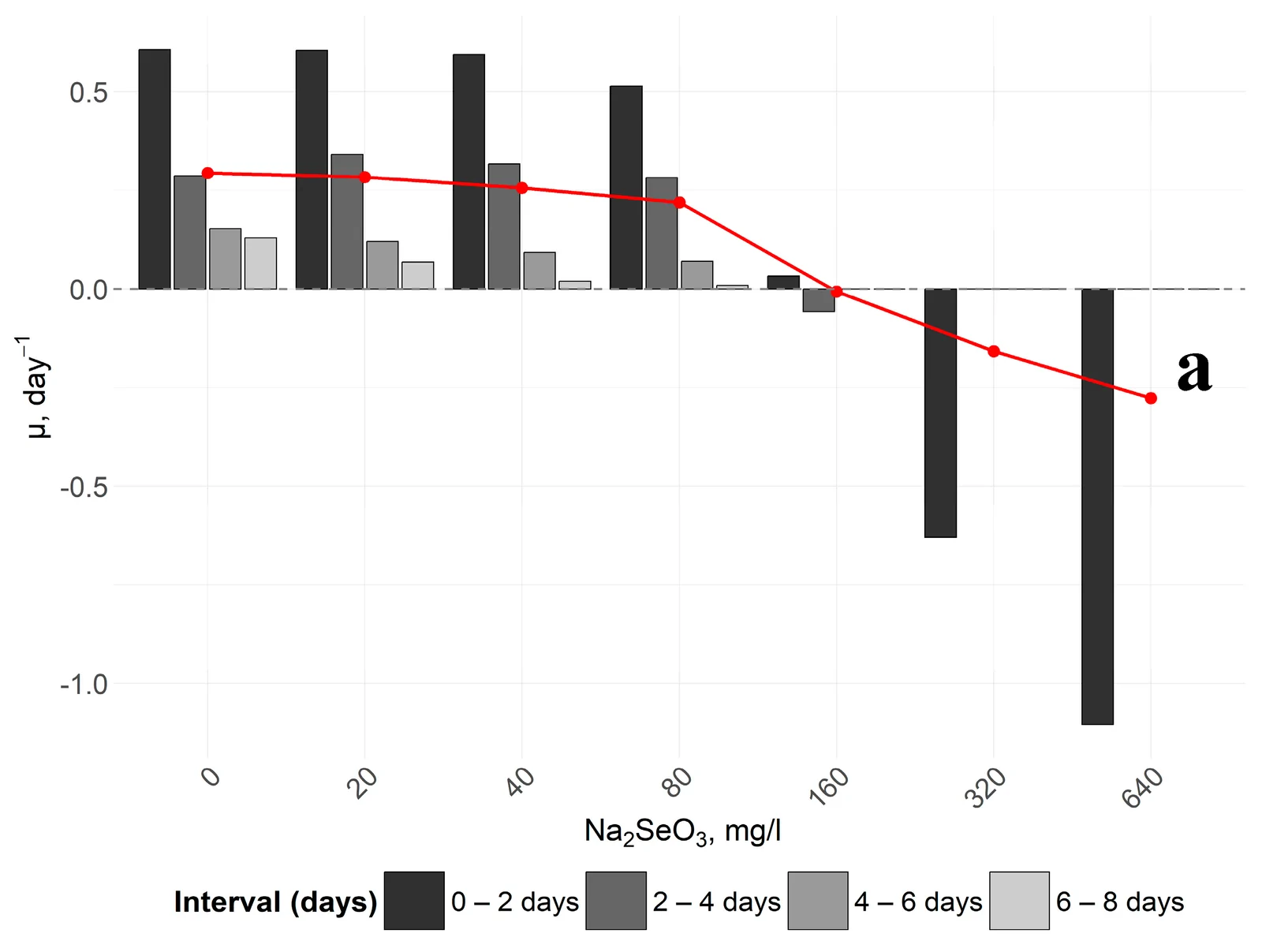

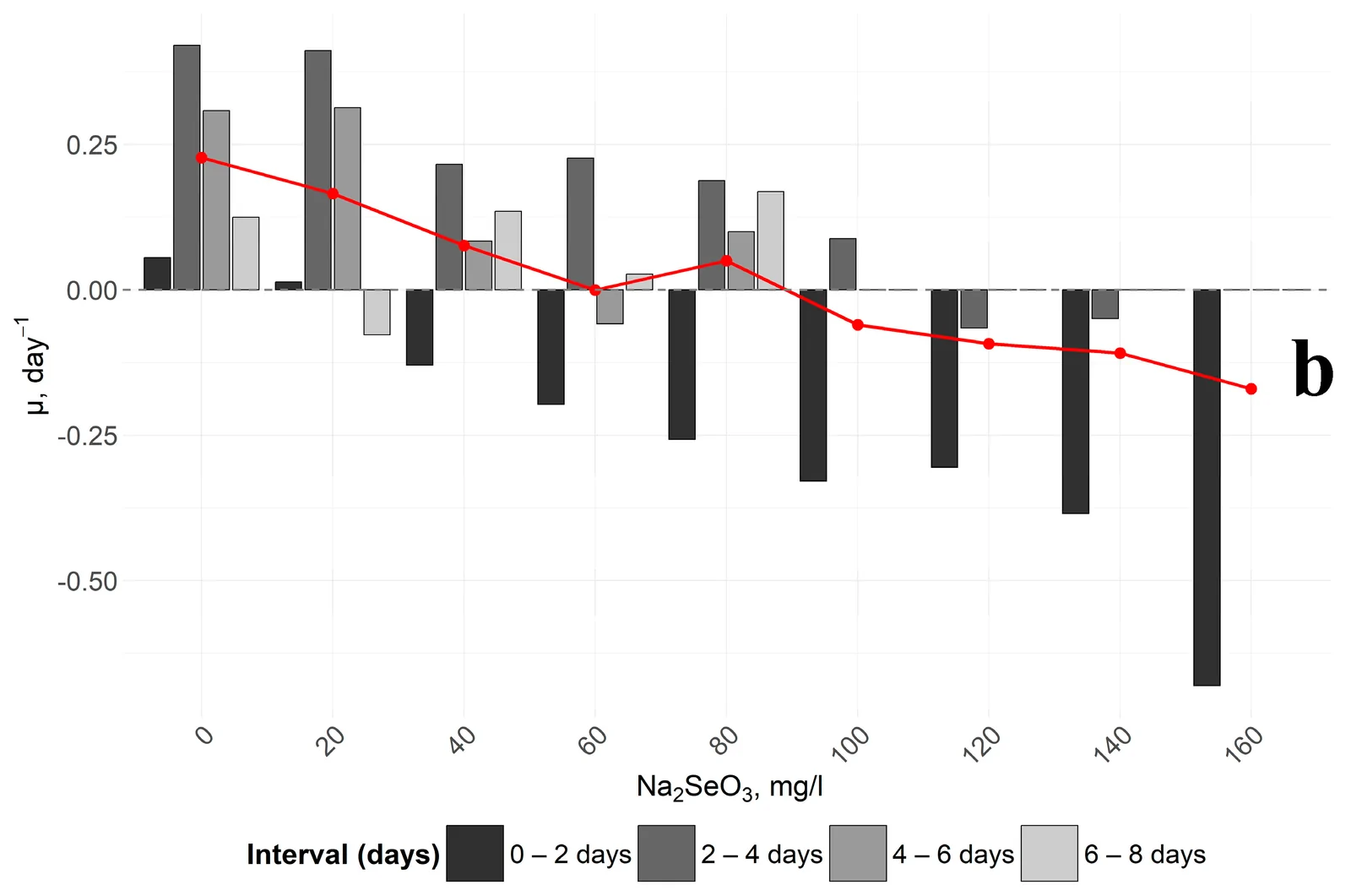
Specific growth rates by intervals (0–2, 2–4, 4–6, 6–8 days) and specific growth rate averaged over four time intervals (red line) at different sodium selenite concentrations: (**a**) first experiment, (**b**) second experiment (excluding negative values for both experiments). Results are presented as descriptive means without error bars.

The specific growth rate calculated using Equation (1), and the average daily biomass productivity rate calculated using OLS, for the first and second experiments are presented in Appendix C.1. Figure A1 and Figure 6, respectively. In the first experiment, the highest average specific growth rate and average daily biomass productivity rate are observed in the control (0 mg/L) and at 20 mg/L of selenite (μ ≈ 0.29–0.28 day^−1^, productivity rate ≈ 289–319 mg/L·day). As the concentration increases to 40–80 mg/L, the growth rate drops by a factor of 1.1–1.7 (Figure A1). At concentrations ≥ 160 mg/L, μ becomes negative, confirming the toxic effect and culture death in the early stages.

In the second experiment, the highest growth rate is observed in the sample without additional selenite supplementation, where μ = 0.227 day^−1^ and the biomass productivity rate is 239 mg/L·day (Figure 6). At a sodium selenite concentration of ≥40 mg/L, the growth rate drops sharply, and at ≥100 mg/L, μ becomes negative, accompanied by rapid culture death.

Detailed statistical analysis is provided in Appendix A.3. Statistical analysis of biomass productivity rates revealed a highly significant inhibitory effect of selenite concentration on average daily biomass increase in both experiments. The first concentration causing significant inhibition was 40 mg/L in both experiments. Concentrations of 0 and 20 mg/L formed a statistically homogeneous group, whereas concentrations of 40 mg/L and above were significantly different from the control with significantly slower biomass growth.

### 2.5. Changes in pH of Culture Medium

**Figure 7:**
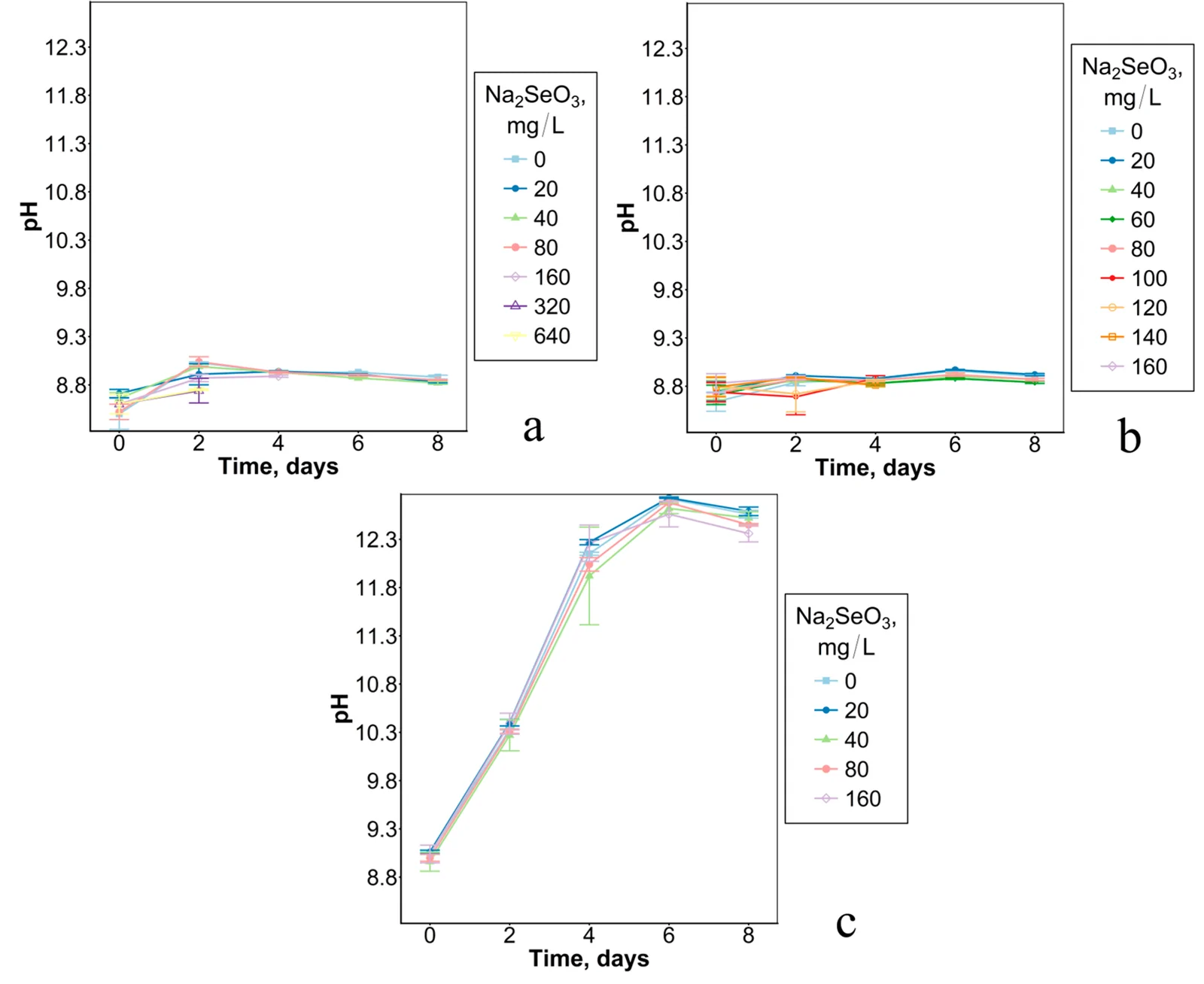
Dependence of sample pH values on sodium selenite concentrations in the nutrient medium: (**a**) experiment 1, (**b**) experiment 2, (**c**) experiment 3. Error bars represent SE of mean.

The pH change (Figure 7) in the first experiment reflects the pH dynamics over time for the specified sodium selenite concentrations. Initial pH values were similar across all groups (8.50–8.71 on day 0), rising to 8.74–9.04 by day 2. Subsequently, the pH either stabilized or decreased slightly. For high concentrations (160–640 mg/L), data are limited to days 0–2 due to culture death, with the pH remaining in the range of 8.59–8.87.

In the second experiment, the pH change graph shows initial values of 8.64–8.83 (day 0), increasing to 8.69–8.87 by day 2. Thereafter, the pH stabilized. For concentrations ≥ 100 mg/L, data are limited until the point of culture death, with pH values ranging between 8.81 and 8.88. Overall, the pH dynamics in the second experiment are like those in the first experiment, the pH remains within the alkaline range.

The overall pH stability (changes < 0.5 units) under 3% CO_2_ is explained by the equilibrium between the culture medium and the CO_2_-enriched atmosphere. A control experiment with cell-free Zarrouk medium aerated with 3% CO_2_ (Appendix B) confirmed that CO_2_ aeration alone does not acidify the medium, as the system equilibrates at the given CO_2_ partial pressure without significant pH shifts.

In the third experiment (atmospheric CO_2_), the pH dynamics showed initial values of 8.86–9.14 (day 0), increasing to 10.11–10.50 by day 2. By day 4, pH rose to 11.42–12.45, and by days 6 and 8 it reached 12.43–12.75. For concentrations ≥ 80 mg/L, some data were limited due to culture death; however, even in these variants pH remained high (on day 8, 12.28–12.63). Overall, the pH dynamics in the third experiment were fundamentally different from the first two: a pronounced continuous rise into the strongly alkaline range (up to 12.7) was observed. This increase is attributed to two combined factors: active bicarbonate consumption by the culture during photosynthesis, and physical stripping of dissolved CO_2_ by air bubbling, which shifts the equilibrium HCO_3_^−^ → CO_2_↑ + OH^−^ and drives pH upward even without algal activity. A precise quantitative separation of these two contributions requires additional experiments, which were beyond the scope of this study. Unlike the experiments with 3% CO_2_, where pH stabilized in the range of 8.6–9.0 due to equilibrium with the CO_2_-enriched atmosphere, under atmospheric CO_2_ the continuous stripping of CO_2_ shifts the equilibrium HCO_3_^−^ → CO_2_↑ + OH^−^, allowing pH to rise without limitation.

Detailed statistical analysis is provided in Appendix A.4. Statistical analysis of culture medium pH revealed that higher selenite concentrations caused measurable acidification over time compared to control. In Experiment 1, significant pH reduction was observed from day 2 onward, with the threshold concentration decreasing over time (from 640 mg/L on day 0 to 20 mg/L on day 8). In Experiment 2, significant pH reduction occurred on days 6 and 8 at selenite concentrations of 60 mg/L and above. Overall, the effect became more pronounced with longer exposure, suggesting a time-dependent metabolic stress response under selenite exposure.

### 2.6. Dynamics of Phosphate Content Changes

**Figure 8:**
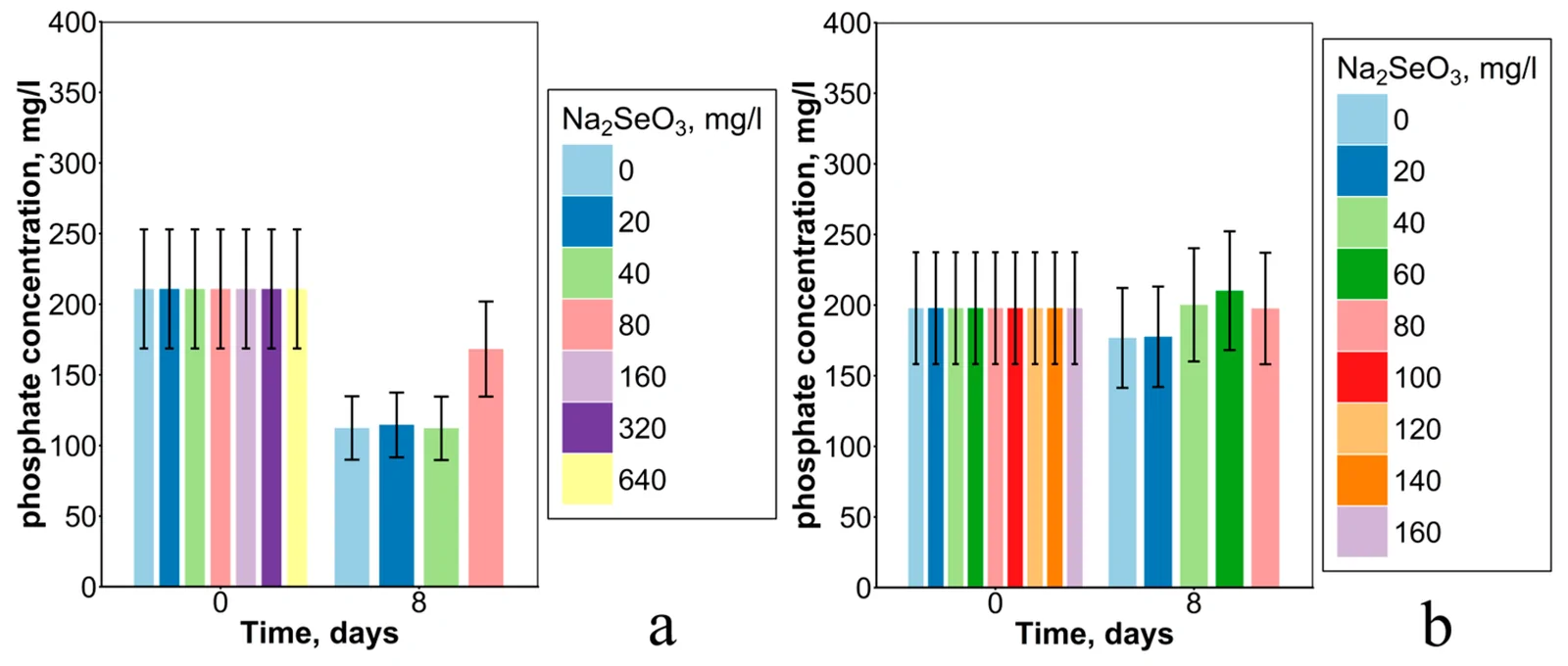
Dependence of phosphate concentration on sodium selenite concentrations in the nutrient medium: (**a**) experiment 1, (**b**) experiment 2. Error bars represent the relative error of the test kit.

The graph of phosphate concentration changes (Figure 8) shows a decrease in phosphate levels (mg/L) over time for sodium selenite concentrations of 0–160 mg/L. Phosphate consumption correlates with biomass growth: the most significant reduction is observed at low selenium doses (where growth is most active). In contrast, at concentrations of 160 mg/L and higher, phosphates are consumed more slowly due to metabolic suppression. In the later stages, a partial return of phosphates to the medium is observed, likely due to cell lysis. Although the nutrient medium composition was identical at the start of both experiments, the measured Day 0 phosphate and nitrate concentrations showed minor variation between replicates, most likely due to analytical measurement error; for each experiment, Day 0 values were therefore replaced by the mean calculated after excluding clear outliers.

In the second experiment, phosphate consumption is lower than in the first, with a less pronounced decrease during active growth. Upon culture death (at concentrations ≥ 100 mg/L), phosphate levels remain high. The relative error for the test kit is 20%.

The nominal phosphate content of the nutrient medium, calculated from its K_2_HPO_4_·3H_2_O content (0.66 g/L), corresponds to approximately 275 mg/L PO_4_^3−^; the measured initial (Day 0) concentration (~200 mg/L) is somewhat lower than this nominal value, which may be explained by partial precipitation of phosphate as insoluble calcium/magnesium phosphate in the presence of Ca^2+^ and Mg^2+^ ions in the medium, reducing the soluble (measurable) fraction.

In the third experiment (atmospheric CO_2_), phosphate consumption was not detectable by the end of the cultivation period.

### 2.7. Changes in Nitrate Content

**Figure 9:**
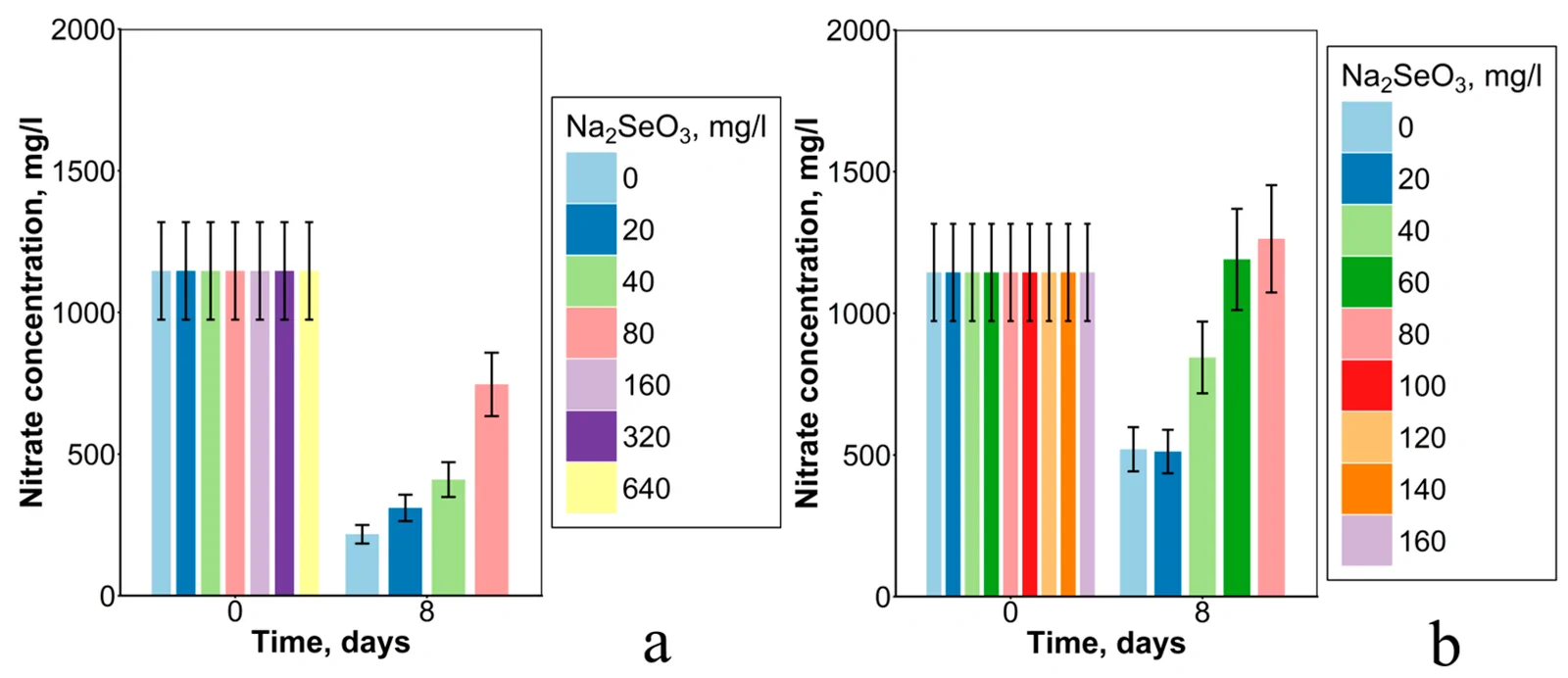
Dependence of nitrate concentration on sodium selenite concentrations in the nutrient medium: (**a**) experiment 1, (**b**) experiment 2. Error bars represent the relative error of the test kit.

Changes in nitrate content during the first and second experiments are shown in Figure 9. The graph indicates a consistent decrease in nitrate concentration. Nitrates are intensively consumed during active growth (low selenium doses), with minimal residual levels in control. During inhibition (80–160 mg/L), consumption slows down, indicating a reduction in the nitrogen metabolism of spirulina under the influence of selenite.

Nitrate consumption in the second experiment is weaker than in the first; upon culture death (≥80 mg/L), nitrate levels remain high or even increase. The correlation with growth is maintained. The relative error for the test kit is 15%.

Concentrations were expressed as NO_3_^−^, mg/L. The nominal nitrate content calculated from the KNO_3_ content of the medium (3.0 g/L) corresponds to approximately 1840 mg/L NO_3_^−^; the measured initial concentration (~1100 mg/L) was lower, which is attributed to the dilution required to bring samples into the calibration range of the method, introducing additional measurement uncertainty.

In the third experiment (atmospheric CO_2_), the consumption of nitrates was not pronounced by the end of the 8-day cultivation period. This lack of significant nutrient uptake is directly attributable to the culture death and the consequent cessation of active metabolism, rather than to selenite toxicity.

### 2.8. Dynamics of Ammonium Ion Concentrations in the Nutrient Medium

In the first experiment, on day 0, the concentration was 0 mg/L in all samples. By day 4, ammonium appeared in all samples. By day 8, ammonium appeared and accumulated in the range of 12.9–25.7 mg/L. The highest values are noted in variants with slowed or inhibited growth, especially at 40–80 mg/L of selenite.

In the second experiment, ammonium (NH_4_^+^) was absent on day 0. By day 4, it appeared in all samples within the range of 38.9–85.6 mg/L, with the maximum observed in the 20 mg/L sample. By day 8, accumulation intensified in samples with slowed growth.

In samples where culture death occurred (≥80 mg/L), data are limited to the early days, but elevated ammonium levels were already present by day 4. High ammonium accumulation in samples with inhibited growth indicates a decrease in the ammonium uptake rate by the spirulina itself.

### 2.9. Changes in Carbonates and Bicarbonates

**Figure 10:**
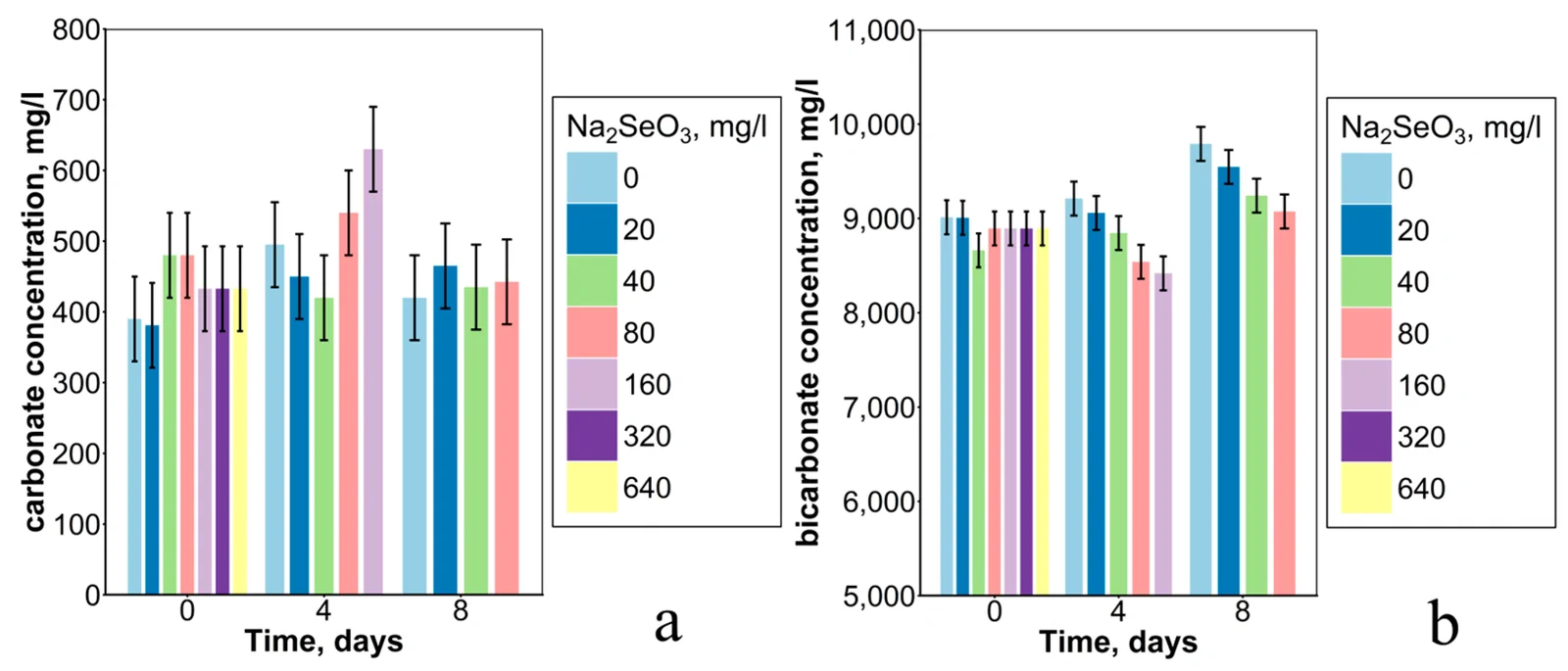
Dependence of the concentration of (**a**) carbonates, (**b**) bicarbonates on sodium selenite concentrations in the nutrient medium in experiment 1. Error bars represent an analytical error.

Graphs showing changes in carbonates (CO_3_^2−^) and bicarbonates (HCO_3_^−^) in the first experiment (Figure 10) indicate that the CO_3_^2−^ concentration increased from initial values and stabilized by Day 8 in samples with active growth. The HCO_3_^−^ concentration decreased and then partially recovered by Day 8 in the viable variants.

Carbonates accumulate during cultivation as a consequence of the pH increase driven by photosynthetic bicarbonate consumption and CO_2_ removal from the medium, which shifts the carbonate equilibrium towards CO_3_^2−^. The dynamics are weakly dependent on selenite, but under inhibition (high doses), changes are minimal due to reduced metabolic activity.

In the second experiment, initial concentrations of carbonates and bicarbonates varied depending on the sample. By Day 4, the following changes were observed:

- Carbonates (CO_3_^2−^) decreased or remained stable, with a slight increase in concentration in some samples;
- Bicarbonates (HCO_3_^−^) decreased, with the most notable consumption occurring in samples with active growth (0 and 20 mg/L).

By Day 8, in viable samples:

- Carbonates (CO_3_^2−^) stabilized or slightly increased;
- Bicarbonates (HCO_3_^−^) partially recovered or stabilized.

In samples where culture death occurred (≥100 mg/L), data are limited to the early days, and changes in the carbonate system were minimal.

**Figure 11:**
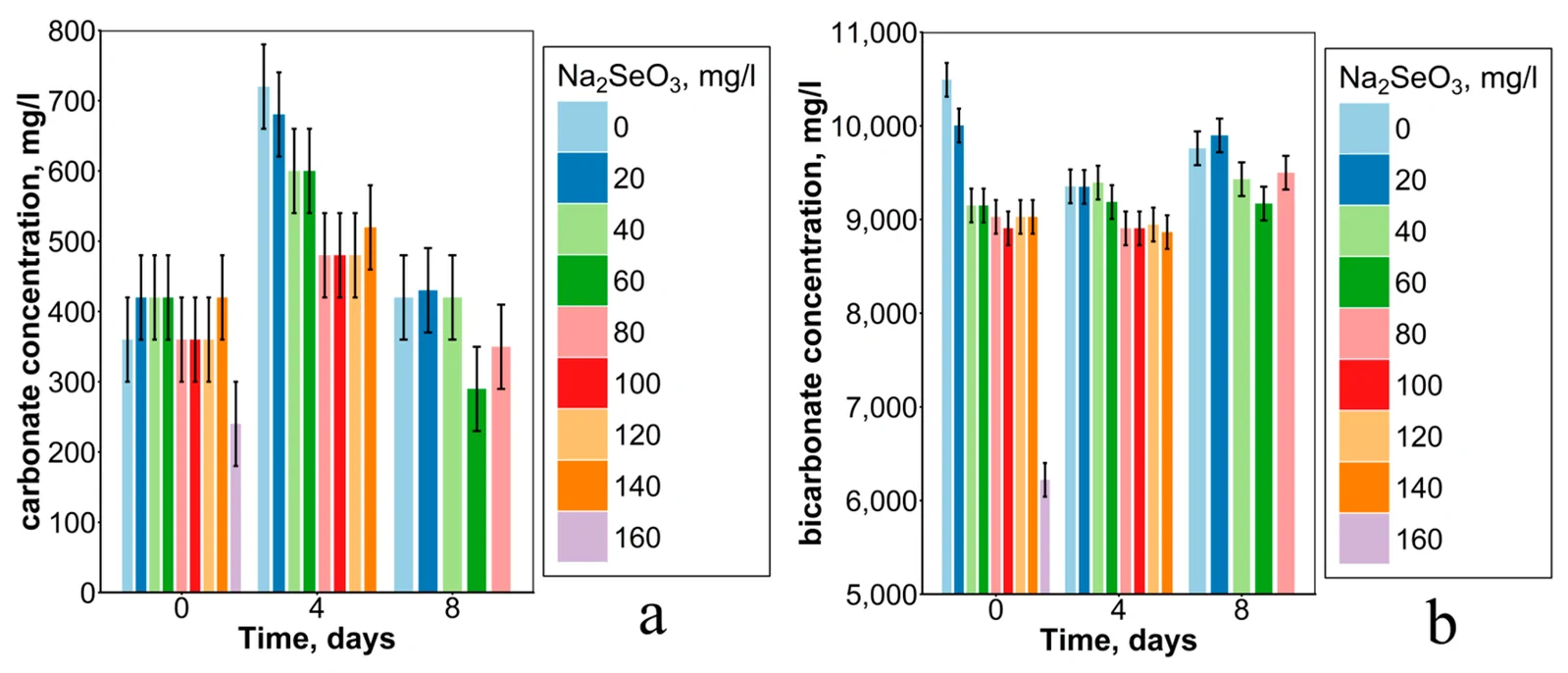
Dependence of the concentration of (**a**) carbonates, (**b**) bicarbonates on sodium selenite concentrations in the nutrient medium in experiment 2. Error bars represent an analytical error.

In the second experiment, the dynamics of carbonates and bicarbonates (Figure 11) were like those in the first: carbonates tended to accumulate or stabilize, while bicarbonates were consumed more actively in samples showing robust growth (nominally 0–20 mg/L). However, the overall consumption of HCO_3_^−^ was less pronounced than in the first experiment, correlating with the lower productivity of the adapted culture. Under growth inhibition conditions (≥60 mg/L), changes in the carbonate system became minimal due to the suppression of photosynthesis and CO_2_ fixation. The measurement error for carbonates and bicarbonates is estimated based on the titration process error at ±60 mg/L and ±180 mg/L, respectively.

**Figure 12:**
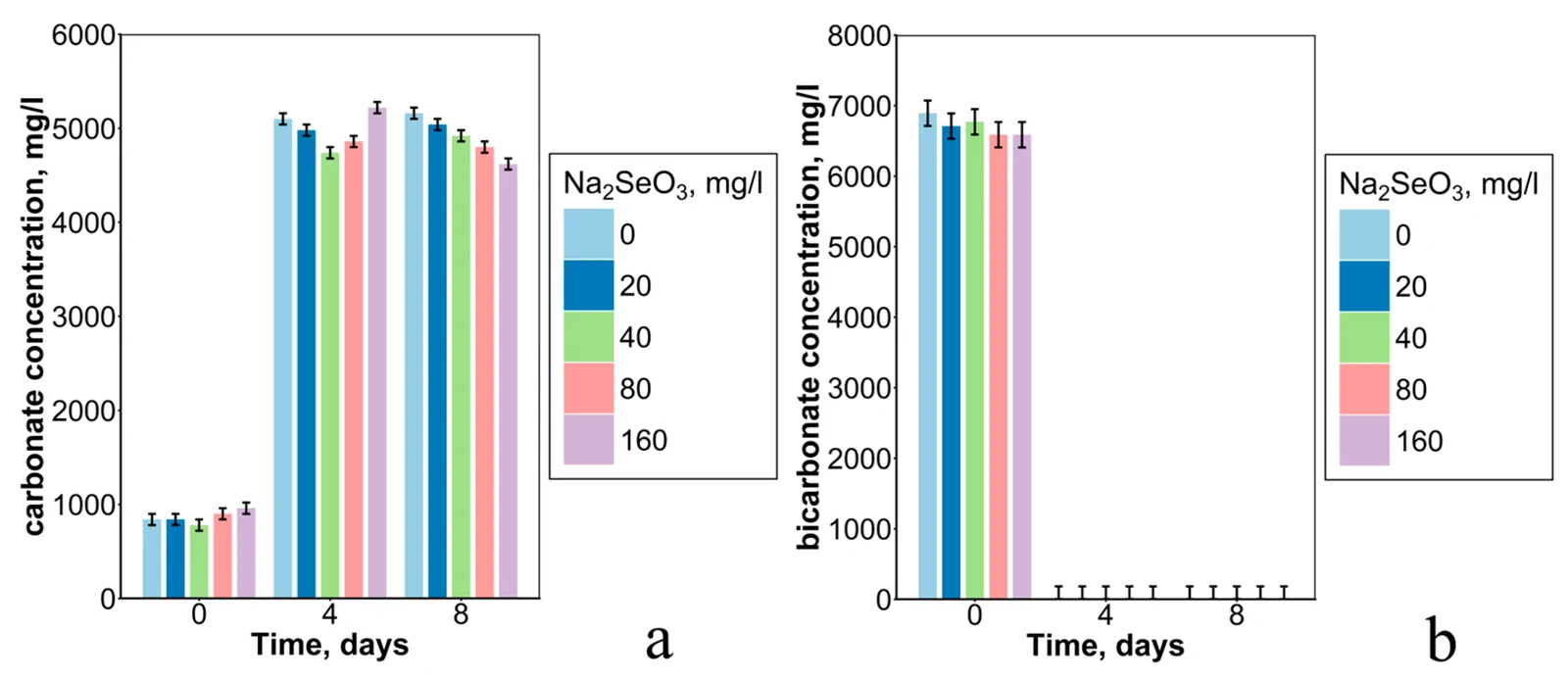
Dependence of the concentration of (**a**) carbonates, (**b**) bicarbonates on sodium selenite concentrations in the nutrient medium in experiment 3. Error bars represent an analytical error.

In the third experiment, the dynamics of carbonates and bicarbonates (Figure 12) followed a completely different pattern from the first two experiments. Carbonates increased sharply from day 0 to day 4, reaching values above 5000 mg/L, and remained at similarly high levels through day 8—far exceeding the carbonate concentrations observed in the earlier experiments. In contrast, bicarbonates fell to zero by day 4 and stayed at zero on day 8, indicating complete depletion of the bicarbonate pool. This suggests that the carbonate system was strongly shifted toward carbonate accumulation, possibly due to intensive CO_2_ fixation or a change in the medium’s initial carbonate-bicarbonate equilibrium. At selenite concentrations above 40 mg/L, carbonate levels remained high, implying that photosynthetic activity was not suppressed, but bicarbonate was exhausted regardless of the selenite dose. The measurement error for carbonates and bicarbonates is estimated based on the titration process error at ±60 mg/L and ±180 mg/L, respectively.

### 2.10. Changes in CO_2_ Concentration

**Figure A2:**
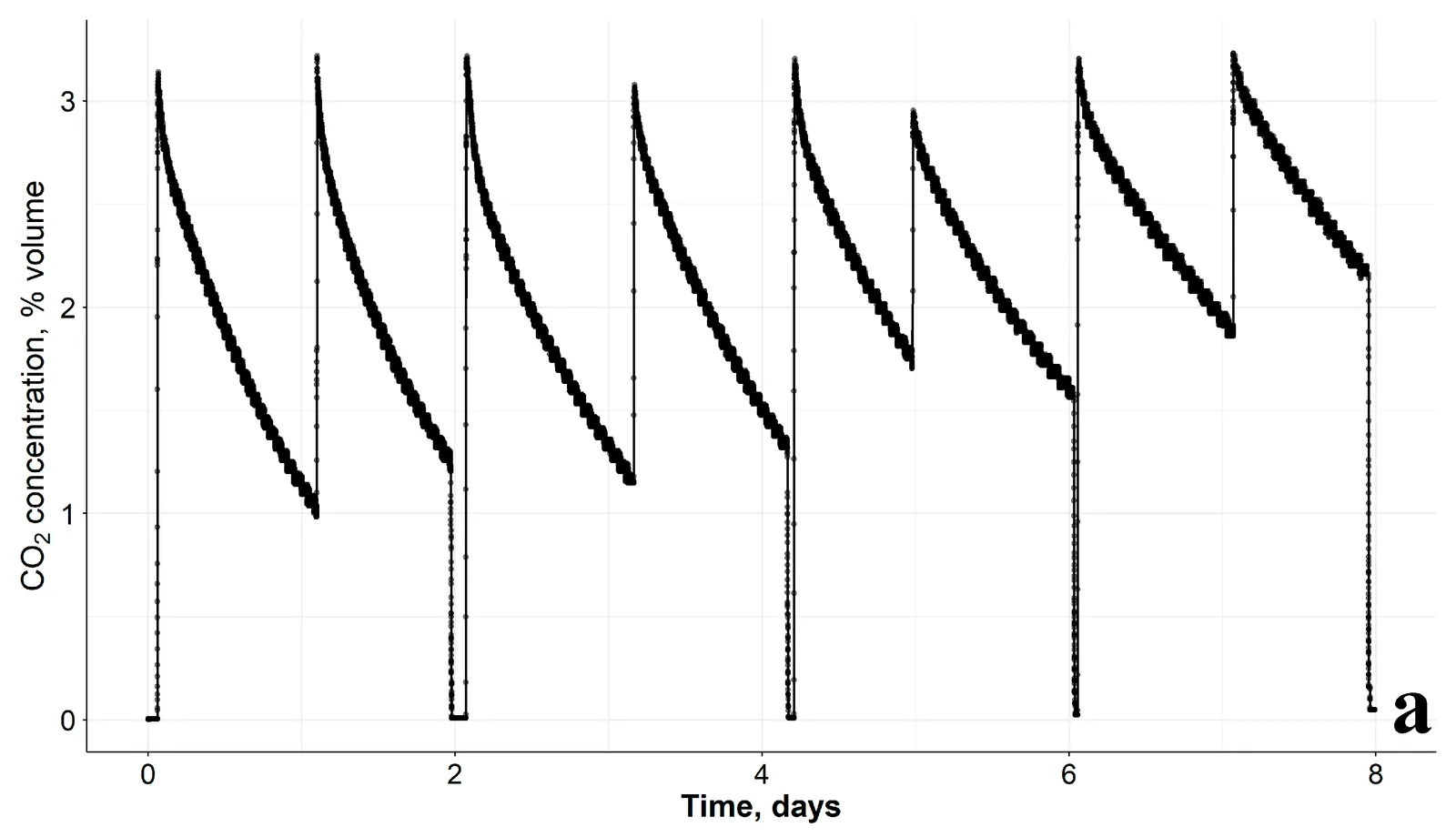

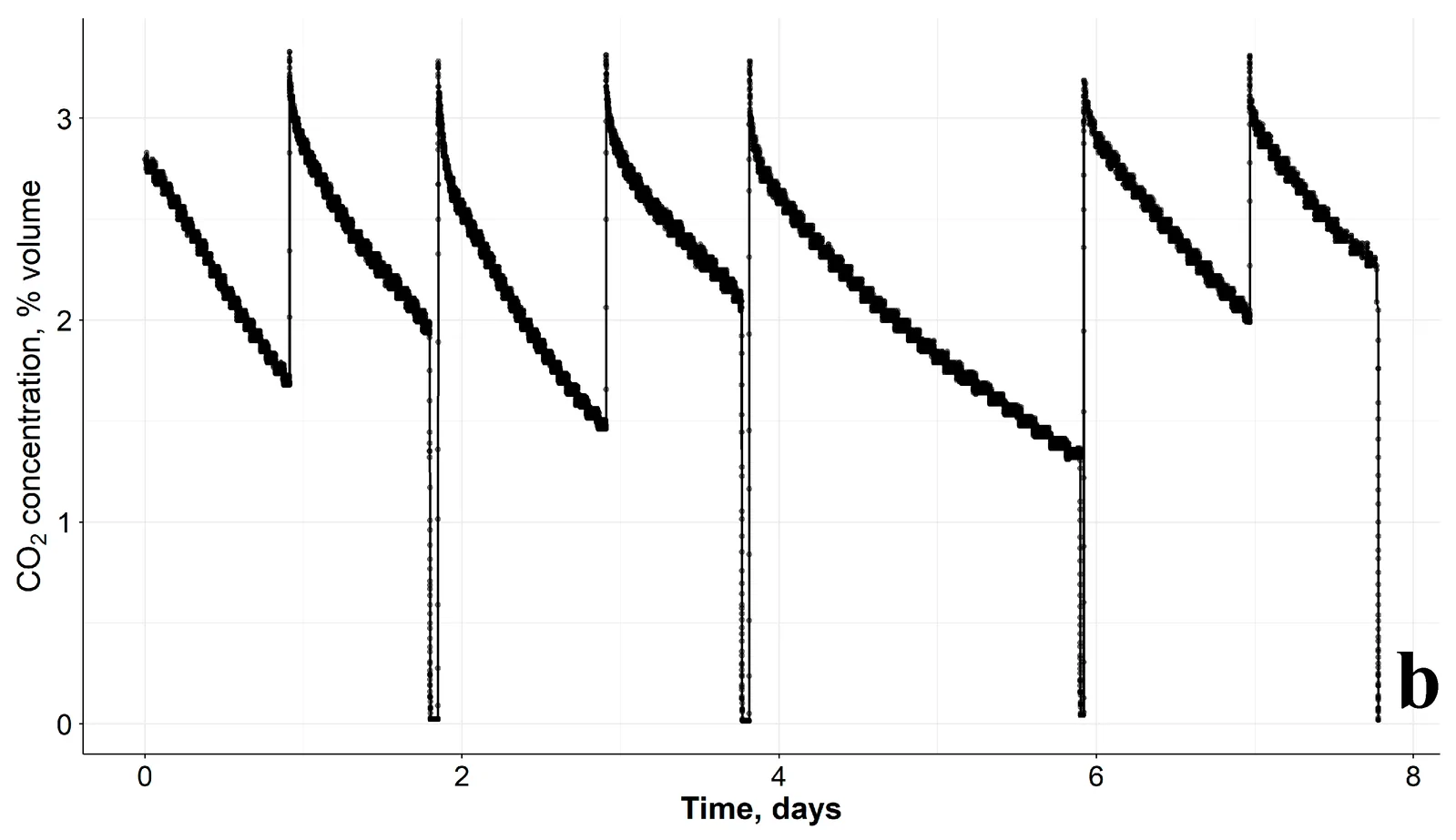
Fluctuations in CO_2_ concentration over time in (**a**) experiment 1 and (**b**) experiment 2.

The concentration of carbon dioxide (CO_2_, vol.%) within the sealed 12 m^3^ gas chamber was recorded in real-time using a gas analyzer in both experiments (Appendix C.2. Figure A2). The CO_2_ measurements were performed only for monitoring the chamber atmosphere, not for evaluating individual treatment effects. In the first experiment, data covered ~194 h (8.09 days) with a sampling frequency of 1–5 min (27,426 records); in the second experiment, it covered ~187 h (7.78 days) with a similar frequency (20,620 records).

In the first experiment, the initial CO_2_ concentration was ~0.0035–0.0039 vol.% (atmospheric background) during the first 1.2 h. Subsequently, it was regulated via periodic gas supply to ~3 vol.% every 24 h to compensate for CO_2_ uptake by the *Limnospira platensis* culture. Following each injection, a gradual decline was observed at an average rate of ~0.01–0.02 vol.%/h. The daily decrease in concentration (consumption by cyanobacteria) averaged 0.5–1 vol.%. Sharp drops to near-zero levels were recorded approximately every two days, coinciding with sampling procedures, after which controlled CO_2_ introduction restored the concentration to the target level of ~3 vol.%.

In the second experiment (using the adapted culture), the initial concentration was ~2.75–2.8 vol.%. The dynamics were consistent with the first trial: gas supply up to ~3 vol.% daily followed by a decline of ~0.01–0.02 vol.%/h. The daily drawdown also averaged 0.5–1 vol.% (falling from ~3 vol.% to ~2–2.5 vol.% in the 24 h prior to the next refill). Sharp drops to zero every two days were attributed to chamber ventilation during sampling. By the conclusion of the experiment, the concentration stabilized at a low level (0.02–0.19 vol.%).

In both experiments, CO_2_ dynamics followed a cyclic pattern: regular replenishment to ~3 vol.% on a daily cycle followed by culture consumption at a rate of ~0.5–1 vol.%/day, punctuated by sharp drops during sampling-related ventilation. The adaptation of the culture in the second experiment did not significantly alter the CO_2_ fixation pattern. The measurement error for CO_2_ concentration is ±0.05 vol.%.

## 3. Discussion

Sodium selenite exerts a cytotoxic effect on *Limnospira platensis*. Our findings define the absolute physiological threshold for *L. platensis* under carbon-saturated conditions. Unlike standard studies, this work evaluates the limits of rapid adaptation, showing that metabolic stability is maintained only within a narrow concentration window under high-intensity gas exchange. For the specific strain and cultivation conditions used in this study, the acute toxicity threshold was found to be in the range of 80–100 mg/L. High concentrations of sodium selenite (above 160 mg/L) cause culture death within the first 2–4 days of the experiment. At low concentrations (20 mg/L), growth was inhibited compared to the control.

These findings align with studies where *Limnospira platensis* showed optimal growth characteristics at a sodium selenite concentration of 5 mg/L [23]. However, conflicting data exist; for instance, some reports suggest that *L. platensis* achieves its fastest growth at 120 mg/L [17], while others claim that concentrations up to 400 mg/L can stimulate growth [16].

It is important to note that previously published research typically utilized *Limnospira platensis* cultivated under standard conditions with atmospheric CO_2_ levels. In our study, the strain was pre-adapted to an elevated CO_2_ concentration (3 vol%) and grown under these high-CO_2_ conditions, which likely influenced the outcomes. Adaptation to high CO_2_ levels may involve shifts in the antioxidant defense system and general metabolism, potentially explaining the discrepancies in selenium toxicity thresholds compared to the existing literature.

The atmospheric CO_2_ experiment (Experiment 3) revealed that selenite toxicity was markedly lower than under 3% CO_2_: at 160 mg/L, growth was not completely inhibited, whereas under 3% CO_2_ this concentration caused complete culture death within the first days. This pattern is consistent with the hypothesis that elevated CO_2_ enhances selenite toxicity. As demonstrated in [24], selenite concentrations as low as 30 mg/L inhibited growth of cultures sparged with 2% CO_2_, supporting our interpretation that elevated CO_2_ enhances selenite toxicity. Additionally, the decline in all treatments after day 4 under atmospheric CO_2_ is likely due to carbon limitation. The long-term adaptation of the strain to 3% CO_2_ may have also contributed to the observed differences by altering the physiological response to selenite.

It is possible that the spirulina pre-adapted to 20 mg/L of sodium selenite showed a tendency toward higher resistance to elevated selenium concentrations compared to the non-adapted culture, but this observation requires further verification.

Morphological changes were observed in the *Limnospira platensis* trichomes in samples treated with sodium selenite, specifically cell lysis and a reduction in the number of cells per trichome. At high selenite concentrations, the increase in trichome number is most likely due to fragmentation rather than cell division. Therefore, trichome counts do not reflect actual biomass growth. While literature regarding the impact of sodium selenite on *L. platensis* morphology is scarce, a similar effect on culture health has been described in studies investigating the impact of heavy metals on the growth of *L. platensis* [25].

Nutrient consumption depends on the viability of the *Limnospira platensis* culture. Nitrates and phosphates are actively consumed during high growth stages (selenite concentrations of 0–20 mg/L), indicating normal nitrogen and phosphorus metabolism. At concentrations of 40–60 mg/L, consumption slows down, and at high concentrations (above 80 mg/L), it almost completely ceases, pointing to the suppression of key metabolic pathways. In the second experiment, the total consumption of nitrates and phosphates was lower than in the first. These differences in nitrate and phosphate consumption between concentration groups were confirmed to be statistically significant (one-way ANOVA with Tukey’s and Dunnett’s post hoc tests, *p* < 0.05; detailed results are provided in Appendix A.3 and Appendix A.4). Differences not supported by statistical testing are not discussed as meaningful trends. This indicates that the inhibitory effect of selenite on nitrate and phosphate uptake is largely indirect, mediated through the general suppression of growth and viability, rather than reflecting a selenite-specific disruption of nitrogen or phosphorus assimilation pathways.

Ammonium is absent from the medium at the start of the experiment but accumulates toward the end, with the highest concentration of ammonium ions observed in samples with high sodium selenite content. This can be explained by the release of intracellular nitrogen from ruptured cells, as well as reduced nitrogen assimilation in the inhibited samples.

In aqueous media, dissolved inorganic carbon (DIC) exists as a mixture of CO_2_ (including carbonic acid), HCO_3_^−^, and CO_3_^2−^. The relative abundance of these species is governed by pH: at alkaline pH (8–9), the system is dominated by HCO_3_^−^, while at higher pH (>10), CO_3_^2−^ becomes the major form [26]. In Zarrouk medium with an initial NaHCO_3_ concentration of 12 g/L, the pH at the start of experiments was ~8.6–8.8, corresponding to an equilibrium where HCO_3_^−^ is the predominant species. When the medium is sparged with CO_2_-enriched gas (3%), the increased CO_2_ partial pressure shifts the equilibrium towards the dissolved CO_2_ and HCO_3_^−^ forms, while the pH remains stable due to the strong buffering capacity of the system. Conversely, sparging with ambient air (0.04% CO_2_) strips dissolved CO_2_ from the solution, shifting the equilibrium HCO_3_^−^ → CO_2_↑ + OH^−^ and driving pH upward. This physicochemical effect is independent of biological activity and must be considered alongside photosynthetic bicarbonate consumption when interpreting pH dynamics. The interplay between these processes determines the availability of inorganic carbon for photosynthesis and, consequently, the growth performance of *Limnospira platensis* under different CO_2_ regimes.

The difference in growth performance between the 3% CO_2_ experiments (Experiments 1 and 2) and the atmospheric CO_2_ experiment (Experiment 3) can be explained by the availability of dissolved inorganic carbon. Under atmospheric CO_2_ (0.04%), the initial bicarbonate in the Zarrouk medium (12 g/L NaHCO_3_) supports growth up to a biomass concentration of approximately 1.4 g·L^−1^ dry weight (Figure 5c). Beyond this point, the bicarbonate pool becomes depleted, and the low CO_2_ partial pressure in the sparging air cannot replenish the inorganic carbon lost through photosynthesis and physical stripping. As a result, the culture enters carbon limitation, leading to growth arrest and subsequent biomass decline. In contrast, under 3% CO_2_ supplementation, the continuous supply of CO_2_ maintains the dissolved inorganic carbon pool, allowing biomass to accumulate beyond 2 g·L^−1^ without carbon limitation (Figure 5a,b). This explains why growth continued in Experiments 1 and 2, whereas in Experiment 3 it ceased even in the control samples (without selenite), indicating carbon limitation rather than selenite toxicity.

Table 2 presents a summary of literature data on the effect of sodium selenite on the growth rate of *Limnospira platensis* and a comparison with the results of this study. The table shows that the growth rates of the control samples differ significantly depending on the cultivation conditions. This confirms that the cultivation regime is a decisive factor in productivity. The growth rates obtained in the present study are comparably high. That ensures the high sensitivity of our system to external influences and stressors. Consequently, this likely accounts for the more rapid and pronounced manifestation of the sodium selenite effect observed in our research.

## 4. Materials and Methods

### 4.1. Experimental Setup

The experiments were conducted in a sealed 12 m^3^ gas chamber equipped with systems to monitor temperature, humidity, pressure, and gas composition (analyzers for O_2_, CO, CO_2_, NH_3_, CH_4_, SO_2_, NO_2_). The chamber was connected to CO_2_ cylinders to achieve a carbon dioxide concentration of 3 vol%. Every two days, after sampling, the chamber was sealed and flushed with CO_2_ from the cylinders to reach an initial concentration of 3 vol%. Before each sampling, the chamber was opened, allowing the CO_2_ concentration to drop to ambient levels. During the two-day intervals between samplings, no additional CO_2_ was supplied. A detailed description of the chamber is provided in [27].

Inside the chamber, 28 cylindrical glass column-type photobioreactors (PBRs), each with a 10 L volume (80 cm height), were placed on the floor. The PBRs were covered with a 20 cm × 20 cm gauze lid. Aerators located at the bottom of the PBRs were used to bubble the gas-air mixture at a flow rate of 1 L/min. The gas for bubbling was drawn from the chamber atmosphere. The bubbling was continuous. Continuous illumination was provided by LED strips with an intensity of 220 µmol quanta·m^−2^·s^−1^. The flow rate and illumination were identical for all PBRs. The temperature within the PBRs was maintained at 27 ± 1 °C.

### 4.2. Experimental Procedure

Three experiments, each lasting 8 days, were performed. In the first experiment, a strain of *Limnospira platensis* adapted to high CO_2_ concentrations was used as inoculum at an initial optical density (OD_655_) of 0.301 ± 0.017. Sodium selenite (anhydrous, Na_2_SeO_3_) was added at the start of the experiment in concentrations of 20, 40, 80, 160, 320, and 640 mg/L. A total of 28 PBRs were used: 4 control PBRs (no sodium selenite) and 4 PBRs for each selenium concentration. Samples for analysis were taken on days 0, 2, 4, 6, and 8. Optical density (OD) and pH were measured every two days. Nutrient medium composition was analyzed on days 0, 4, and 8. Microscopic analysis of the culture was performed on days 0 and 8. Day 0 sampling was performed 1–2 h after the addition of sodium selenite.

For the second experiment, the inoculum used was biomass from the first experiment previously grown at a sodium selenite concentration of 20 mg/L, with an initial optical density of 0.387 ± 0.056. Sodium selenite was added at the start of the experiment in concentrations of 20, 40, 60, 80, 100, 120, 140, and 160 mg/L, alongside control PBRs. This experiment utilized 27 PBRs: 3 control PBRs and 3 for each selenium concentration. Culture and medium analyses followed the same schedule as the first experiment.

Experiment 3 was performed under the same conditions but with ambient atmospheric CO_2_ (no CO_2_ enrichment), using selenite concentrations of 0, 20, 40, 80, and 160 mg/L, with two PBRs per concentration.

**Figure 13:**
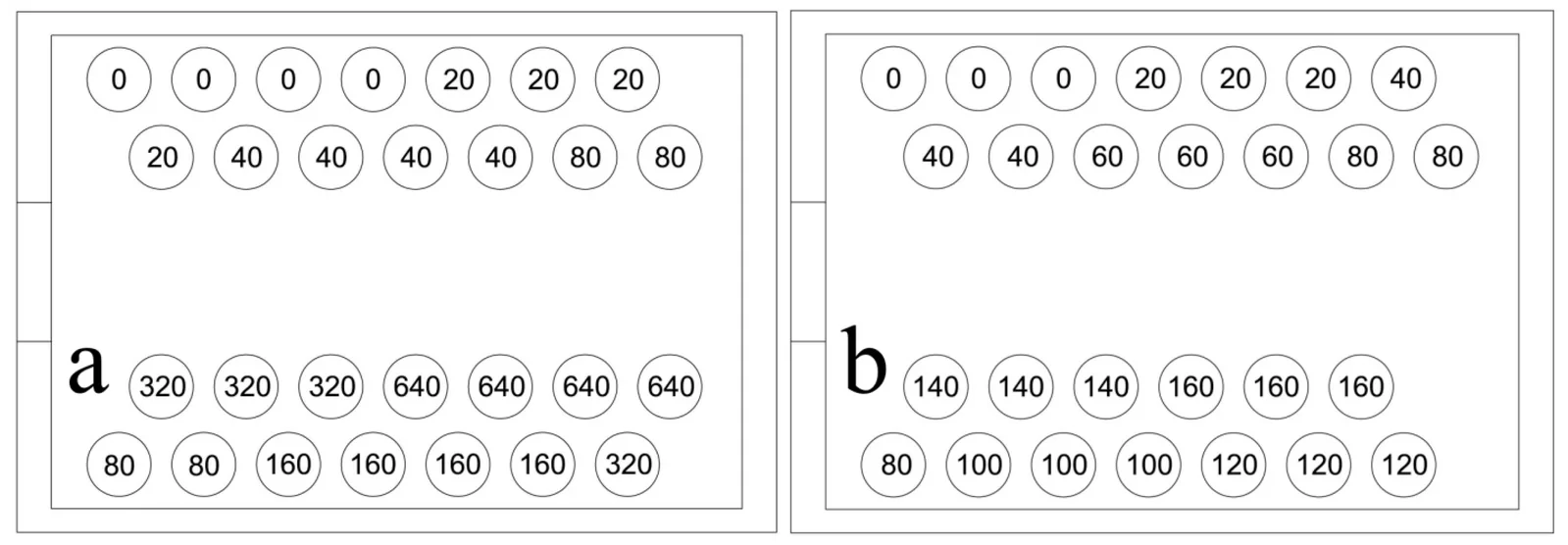
Schematic layout of photobioreactors (PBRs) in the chamber during Experiment 1 (**a**) and Experiment 2 (**b**). Circles represent PBRs; numbers indicate the sodium selenite concentration (mg/L) added at the start. In Experiment 2, the inoculum was biomass from Experiment 1 grown at 20 mg/L sodium selenite.

The layout of the PBRs with different sodium selenite concentrations within the chamber for the first and second experiments is shown in Figure 13.

#### 4.2.1. Adaptation and Cultivation

The experiments utilized a *Limnospira platensis* strain previously adapted to high atmospheric CO_2_ concentrations [28]. The strain was cultivated continuously for 6 months at 3 vol% CO_2_. The original (unadapted) strain *Limnospira platensis* rsemsu P Bios was obtained from the Microalgae Culture Collection of the Faculty of Geography, Lomonosov Moscow State University. The strain is designated as *Limnospira platensis* [29]. A modified Zarrouk medium prepared with distilled water was used: (NaHCO_3_ 12 g/L, KNO_3_ 3.0 g/L, K_2_HPO_4_·3H_2_O 0.66 g/L, K_2_SO_4_ 1.0 g/L, MgSO_4_·7H_2_O 0.2 g/L, NaCl 1.0 g/L, CaCl_2_ 0.04 g/L, FeSO_4_·7H_2_O 0.018 g/L, EDTA 0.08 g/L, Trace elements 1 mL/L).

#### 4.2.2. Filtration

Biomass was harvested on day 8 by filtration through stainless steel mesh filters (80 µm mesh size). The filtrate was analyzed for residual nutrients, and the sediment was analyzed for dry weight. The filtration process for *L. platensis* is gravity-driven due to the large size of the trichomes (up to 800 µm). The filtrate contained less than 0.1 g/L of residual biomass, and the collected biomass was washed with distilled water before drying.

#### 4.2.3. Drying

The filtered biomass was frozen in a lyophilization chamber at −20 °C for 4 h. The frozen samples were then dried at 35 °C for 12 h until a constant weight was achieved.

### 4.3. Research Methods

#### 4.3.1. Determination of Biomass Yield

Biomass yield was calculated as the final dry weight (g/L) of *L. platensis* in PBRs with positive biomass growth.

#### 4.3.2. Determination of Optical Density

Optical density (OD) was measured using an Expert-003 photometer at a wavelength of 655 nm in cuvettes with a 5 mm path length.

#### 4.3.3. pH Determination

The pH of the medium was measured using an Expert-pH meter. Calibration was performed every 7 days using buffer solutions (pH 9.18, 6.86, and 4.01). The measurement error was ±0.01.

#### 4.3.4. Phosphate Determination

Phosphate concentration was determined photometrically using a molybdate reagent (the molybdenum blue method) on an Expert-003 photometer at a wavelength of 700 nm on days 0, 4, and 8. The method is based on the interaction of orthophosphates with ammonium molybdate in an acidic medium to produce phosphomolybdic heteropoly acid. The reduction of this acid results in the formation of a stable blue compound; the intensity of the color is directly proportional to the orthophosphate content (GOST 18309-2014, Method B) [30]. Phosphate measurements were performed using a colorimetric test kit with a specified relative error of 20%. Since these are not replicate-based errors, no formal statistical tests were applied to the phosphate data.

#### 4.3.5. Nitrate Determination

Nitrate concentration was determined photometrically following the reduction of nitrates to nitrites using a catalyst and Griess reagent on an Expert-003 photometer at a wavelength of 525 nm on days 0, 4, and 8. The method is based on the reduction of nitrates to nitrites by metallic magnesium. Subsequently, the nitrites react with the Griess reagent, leading to the formation of dye whose color intensity is proportional to the nitrate concentration (GOST 33045-2014, Method B) [31]. Nitrate measurements were performed using a colorimetric test kit with a specified relative error of 15%. As these are not based on biological replicates, the nitrate data were not subjected to formal statistical testing; we consider only those differences that clearly exceed the 15% error as indicative of meaningful changes.

#### 4.3.6. Determination of NH_4_^+^ Ion

Ion concentrations were determined using ion-selective electrodes (ISE) on an Expert-pH analyzer. NH_4_^+^ concentration was measured in the medium on days 0, 4, and 8 [32].

#### 4.3.7. Determination of Carbonates and Bicarbonates

Carbonates (CO_3_^2−^) and bicarbonates (HCO_3_^−^) were determined via titration on days 0, 4, and 8. The equivalence points were fixed by the color change of phenolphthalein and methyl orange indicators [33].

#### 4.3.8. Microscopy

Microscopy was performed using a Levenhuk MED Series light microscope at magnifications of ×100 and ×400. Culture viability was assessed through differential staining of cells with methylene blue [34]. Sampling was conducted on days 0 and 8. Live and dead trichomes were counted using a Goryaev chamber (hemocytometer) across 10 large squares. A trichome was considered viable if the majority of its cells remained unstained; trichomes with predominantly blue-stained cells were considered non-viable. In addition to pigment presence, morphology and motility were also evaluated.

#### 4.3.9. Gas Composition Analysis

The gas composition (CO_2_, O_2_, etc.) was continuously monitored in the chamber using a gas analyzer. CO_2_ fixation was recorded based on concentration changes in the chamber, with measurements taken every 5–6 s.

#### 4.3.10. Biomass Growth Rate

The final concentration of dry spirulina biomass was calculated using the following formula:

(1) $$C_{spirulina}={OD}_{655}\times 0.85,$$

where 0.85 is the empirical conversion factor (g/L per OD unit). It should be noted that final biomass yields were determined by direct weighing after filtration and drying, while OD was used for monitoring growth dynamics. The conversion factor was applied uniformly across all treatments to ensure comparability.

The specific growth rate (μ, day^−1^) was calculated using the equation:

(2) $$\mu =\frac{\ln\left(\frac{C_{spirulina2}}{C_{spirulina1}}\right)}{\Delta t},$$

for successive intervals (0–2, 2–4, 4–6, and 6–8 days). The average daily increase in $C_{spirulina}$ (mg/L·day) was also determined as Δ$C_{spirulina}$/Δt.

### 4.4. Statistical Analysis

Statistical analysis of experimental data was performed using R software (version 4.5.3) with the packages ggplot2 (version 4.0.2), dplyr (version 1.2.0), car (version 3.1.5), multcomp (version 1.4.30), and multcompView (version 0.1.11). All data are presented as mean ± standard error of the mean. The significance level for all statistical tests (Shapiro–Wilk, Levene’s, two-way and one-way ANOVA, Dunnett’s and Tukey’s post hoc tests, and OLS regression) was set at *p* < 0.05. Normality and homogeneity of variances were checked as a qualitative procedural step (Shapiro–Wilk and Levene’s tests); detailed results are provided in Appendix A. ANOVA is robust to moderate deviations from normality with equal sample sizes, which justified the use of parametric tests. To evaluate the effects of sodium selenite concentration (factor 1) and cultivation time (factor 2), as well as their interaction on spirulina biomass accumulation, pH and alive trichome percentage a two-way analysis of variance (ANOVA) was applied. The model included main effects of both factors and their interaction. ANOVA results are reported with F-values and significance levels (*p*).

To identify differences between selenite concentrations within each time point (days 0, 2, 4, 6, and 8), one-way ANOVA was performed separately for each experiment and each day. When statistically significant effects were detected by one-way ANOVA (*p* < 0.05), multiple pairwise comparisons were conducted using Tukey’s HSD (Honestly Significant Difference) test. Dunnett’s test was used to compare each selenite concentration against the control group (0 mg/L). The test was performed separately for each day of the experiment. Results are presented with estimated differences (Estimate), standard errors, and adjusted *p*-values.

Average daily biomass growth was estimated using ordinary least squares (OLS) linear regression applied to biomass accumulation data over the cultivation period. Only intervals with positive biomass increment between consecutive time points were included in the regression to ensure that growth rates reflected actual proliferation periods. The standard error of the slope was used as a measure of precision for each growth rate estimate. Model fit was assessed using the coefficient of determination (R^2^).

The following thresholds were defined for data interpretation based on the statistical comparisons described above. The overall toxic threshold was defined as the lowest selenite concentration causing a statistically significant decrease in biomass accumulation (dry weight) compared to the control (Dunnett’s test, *p* < 0.05). The acute toxicity threshold was defined as the lowest selenite concentration causing a significant decrease in viability within the first few days of exposure (Tukey’s HSD, *p* < 0.05). The threshold for a significant reduction in viability was defined as the concentration at which the proportion of live trichomes significantly decreased relative to the control on day 8 (Dunnett’s test, *p* < 0.05). The acute sensitivity threshold was defined as the concentration at which early stress indicators (e.g., growth reduction, morphological changes) were first detected (based on ANOVA and pairwise comparisons, *p* < 0.05). Finally, the absolute physiological threshold was defined as the highest selenite concentration allowing any positive growth (or viability > 0%), determined from biomass and viability data.

## 5. Conclusions

It has been established that sodium selenite exerts a dose-dependent cytotoxic effect on *Limnospira platensis* at elevated CO_2_ concentration (3 vol.%). The acute toxicity threshold lies within the range of 80–100 mg/L. At concentrations > 160 mg/L, culture death occurs within the first 2–4 days, and at ≥80 mg/L, the specific growth rate becomes negative.

It was determined that the addition of sodium selenite caused significant pH deviations only at high concentrations. Despite these statistically significant differences, the magnitude of pH change remained within the optimal range for spirulina growth (8.8–9.0), and the overall dynamics were primarily driven by the metabolic activity of the culture rather than directly correlated with selenite concentration.

It was shown that the consumption of nitrates and phosphates strictly correlates with viability and biomass growth. Active consumption occurs at 0–20 mg/L of selenite, slows down at 40–60 mg/L, and practically ceases at ≥80 mg/L. In the adapted culture, overall nutrient consumption is lower.

The dynamics of bicarbonates and carbonates were found to be minimally dependent on sodium selenite concentration due to the use of molecular CO_2_ as the primary carbon source. No significant changes in bicarbonates were observed, and carbonate accumulation was minimal due to the negligible rise in pH.

The results demonstrate that CO_2_ plays a critical role in modulating selenite toxicity and carbon availability in *Limnospira platensis* cultures. Elevated CO_2_ (3%) increases sensitivity to selenite but maintains carbon supply for biomass accumulation, while under atmospheric CO_2_ the toxicity is less pronounced but growth becomes carbon-limited at higher biomass densities. These findings highlight the importance of CO_2_ conditions in designing selenium-enrichment strategies.

For further research, it is recommended to clarify the optimal concentrations of sodium selenite in the range of 0–20 mg/L, study the properties and biochemical composition of the resulting selenium-enriched biomass, conduct longer cultivation experiments (including in continuous mode), and evaluate the bioavailability and biological activity of the product.

## Figures and Tables

**Table 1:** Dry mass of spirulina per photobioreactor (M_1PBR_) and spirulina concentration (C_spirulina_) in the suspension at the end of the experiment (C_Se_—sodium selenite concentration).

1st Experiment			2nd Experiment		
C_Se_, mg/g	M_1PBR_, g	C_spirulina_, g/L	C_Se_, mg/g	M_1PBR_, g	C_spirulina_, g/L
0	27.5	2.88	0	21.7	2.27
20	24	2.52	20	21.3	2.24
40	20	2.09	40	14.1	1.48
80	16.3	1.68	80	7.1	0.74

**Table 2:** Comparative analysis of *Limnospira platensis* growth rates under various sodium selenite concentrations (C_Se_) and cultivation conditions.

Experimental Conditions (T, Light Int., CO_2_, Cult. Duration)	C_Se_, mg/L	Growth Rate, mg/L·day	Source
35 °C, 315.2 µmol quanta·m^−2^·s^−1^, 5 days	0	270	[16]
	0.5	490	
	10	510	
	40	410	
	400	290	
Room temperature, 12 h of light and 12 h of darkness, 14 days	0	34.1	[17]
	21	48.7	
	52	47.8	
	80	48.7	
	121	49.2	
	152	40.2	
28–32 °C, 55–85 µmol quanta·m^−2^·s^−1^, 6 days	0	131	[18]
	25	134	
	50	141	
	75	144	
	100	140	
	125	145	
	150	139	
	175	125	
	200	120	
27 °C, 220 µmol quanta·m^−2^·s^−1^, 8 days, CO_2_ 3 vol.%	0	319	This work (exp. 1)
	20	289	
	40	229	
	80	161	

## Data Availability

The original contributions presented in the study are included in the article material; further inquiries can be directed to the corresponding author.

## Appendix A

### Appendix A.1. Statistical Analysis of Viability

ANOVA was utilized to analyze the numbers of trichomes (dead, alive and total). Normality of the data was confirmed by the Shapiro–Wilk test, and homogeneity of variances was confirmed by Levene’s test, justifying the use of parametric one-way ANOVA followed by Dunnett’s post hoc test for comparisons with the control. In Experiment 1, ANOVA revealed a significant effect of selenite concentration on total trichomes (*F*_3_,_12_ = 7.36, *p* = 0.0045), live trichomes (*F*_3_,_12_ = 9.46, *p* = 0.0017), and dead trichomes (*F*_3_,_12_ = 3.57, *p* = 0.048). Dunnett’s test showed that only the highest concentration (80 mg/L) significantly increased dead trichomes compared to the control (*p* = 0.049). In Experiment 2, ANOVA demonstrated a highly significant effect of selenite on total trichomes (F_4_,_10_ = 10.48, *p* = 0.0006) and live trichomes (F_4_,_10_ = 11.16, *p* = 0.0005). Dunnett’s test identified a significant reduction in total trichomes at 60 mg/L (*p* = 0.034) and in live trichomes at both 60 mg/L (*p* = 0.005) and 80 mg/L (*p* = 0.012). These results confirm a concentration-dependent toxic effect of selenite, with a threshold for significant inhibition observed at 60–80 mg/L.

ANOVA was utilized to analyze the viability of spirulina. Normality of the percentage data was confirmed by the Shapiro–Wilk test, and homogeneity of variances was confirmed by Levene’s test, justifying the use of parametric one-way ANOVA followed by Dunnett’s post hoc test for comparisons with the control. In Experiment 1, ANOVA revealed a significant effect of selenite concentration on the percentage of live trichomes (*F*_3_,_12_ = 4.87, *p* = 0.019). Dunnett’s test showed that the percentage of live trichomes was significantly reduced at 80 mg/L compared to the control (*p* = 0.014). In Experiment 2, ANOVA demonstrated a highly significant effect of selenite on the percentage of live trichomes (F_4_,_10_ = 15.23, *p* = 0.0002). Dunnett’s test identified a significant decrease in live trichomes percentage at 60 mg/L (*p* = 0.002) and 80 mg/L (*p* = 0.001). These results confirm that selenite exposure reduces the proportion of live trichomes in a concentration-dependent manner, with a threshold for significant effect at 60–80 mg/L.

### Appendix A.2. Statistical Analysis of Biomass Concentrations

Normality of biomass concentration data was confirmed by Shapiro–Wilk test for the majority of groups, and homogeneity of variances was confirmed by Levene’s test for most experimental days, supporting the use of parametric methods. Two-way ANOVA revealed highly significant effects of day (F_4_,_81_ = 1648.77, *p* < 0.001), concentration (F_6_,_81_ = 151.18, *p* < 0.001), and their interaction (F_16_,_81_ = 45.65, *p* < 0.001) in Experiment 1, and similarly significant effects in Experiment 2 (day: F_4_,_72_ = 59.86, *p* < 0.001; concentration: F_8_,_72_ = 26.97, *p* < 0.001; interaction: F_23_,_72_ = 7.145, *p* < 0.001). One-way ANOVA performed separately for each day demonstrated significant concentration-dependent effects from day 2 onward in both experiments. In Experiment 1, Dunnett’s test identified a significant reduction in biomass at 80 mg/L and higher concentrations from day 2 (*p* = 0.0095) through day 8 (*p* < 0.001).

In Experiment 1 Dunnett’s test revealed that the first concentration causing significant biomass reduction compared to the control was 640 mg/L on day 0 (*p* = 0.035), 80 mg/L on day 2 (*p* = 0.0095), 80 mg/L on day 4 (*p* = 0.033), 40 mg/L on day 6 (*p* = 0.024), and 20 mg/L on day 8 (*p* < 0.001). These results demonstrate a progressive decrease in the toxic threshold over time, with even low concentrations becoming inhibitory by the end of the experiment. In Experiment 2, the first concentration causing significant biomass reduction was 160 mg/L on day 0 (*p* = 0.0073). From day 2 onward, the threshold decreased to 40 mg/L, with significant effects observed on day 2 (*p* < 0.001), day 4 (*p* < 0.001), day 6 (*p* < 0.001), and day 8 (*p* = 0.013). Unlike Experiment 1, the threshold remained stable at 40 mg/L after day 2, indicating a consistent toxic response once the initial lag phase was overcome. Tukey HSD post hoc analysis further confirmed that concentrations ≥ 20 mg/L in Experiment 1 and ≥40 mg/L in Experiment 2 formed distinct statistical groups differing significantly from the control.

The significant reduction in biomass concentration on day 0 at the highest selenite doses (640 mg/L in Experiment 1 and 160 mg/L in Experiment 2) is attributed to an acute physiological shock, as measurements were taken 1–2 h after selenite addition. During this interval, rapid cell lysis and bleaching were observed, leading to a detectable drop in optical density even at the initial time point.

### Appendix A.3. Statistical Analysis of Biomass Productivity Rate

Statistical analysis of OLS biomass productivity rate data excluding negative growth intervals showed a highly significant effect of selenite concentration on average daily biomass increase in both experiments, as revealed by one-way ANOVA (Experiment 1: *F*_4_,_15_ = 87.43, *p* < 0.001; Experiment 2: *F*_5_,_14_ = 18.71, *p* < 0.001). OLS (Ordinary Least Squares) regression was applied to estimate growth rates from biomass accumulation over time, yielding high coefficients of determination (R^2^ = 0.90–1.00 for Experiment 1 and R^2^ = 0.86–1.00 for Experiment 2, with the exception of 60 mg/L in Experiment 2 where R^2^ = 0.42), indicating that the linear model provided an appropriate fit for most concentrations. Dunnett’s test identified the first concentration causing significant inhibition at 40 mg/L in both experiments (*p* = 0.001 and *p* < 0.001, respectively), while Tukey HSD grouping demonstrated that concentrations of 0 and 20 mg/L formed a statistically homogeneous group (group a), whereas concentrations ≥ 40 mg/L were significantly different from the control.

### Appendix A.4. Statistical Analysis of Culture Medium pH

Testing confirmed that the data met the requirements for parametric analysis. Shapiro–Wilk tests indicated that the majority of groups (88% in Experiment 1, 91% in Experiment 2) were normally distributed. Levene’s test revealed homogeneity of variances for all days except day 8 in Experiment 1 (*p* = 0.021). Two-way ANOVA was therefore considered appropriate for the analysis of pH data.

Two-way ANOVA revealed a significant main effect of selenite concentration on pH in Experiment 1 (*F*_6_,_75_ = 8.93, *p* < 0.001), while the effect of time was not significant. In Experiment 2, a significant main effect of time was observed (*F*_4_,_68_ = 2.60, *p* = 0.046), but no significant effect of concentration. The general trend showed an initial increase followed by stabilization in the alkaline range (8.8–9.0), which is optimal for spirulina growth.

One-way ANOVA showed significant differences among concentrations on days 2 (*p* = 0.015), 4 (*p* = 0.005), 6 (*p* < 0.001), and 8 (*p* = 0.023) in Experiment 1. Dunnett’s test identified the first concentrations causing significant pH reduction: day 0, 640 mg/L (*p* = 0.035); day 2, 320 mg/L (*p* = 0.029); day 4, 160 mg/L (*p* = 0.009); day 6, 40 mg/L (*p* < 0.001); and day 8, 20 mg/L (*p* = 0.038). In Experiment 2, significant concentration-dependent effects emerged on days 6 (*p* = 0.016) and 8 (*p* = 0.014), with Dunnett’s test confirming that 60 mg/L significantly lowered pH relative to the control on both days (*p* = 0.027 and *p* = 0.030, respectively). Overall, higher selenite concentrations (≥320 mg/L in Experiment 1 and ≥60 mg/L in Experiment 2) caused measurable acidification, with the effect becoming more pronounced over time, suggesting a time-dependent metabolic stress response under selenite exposure.

## Appendix B

**Table A1:** pH values (days 0–2) in PBRs without spirulina under different CO_2_ levels.

№ PBR	pH_0_	pH_1_	pH_2_
1	8.458	8.639	8.486
2	8.538	9.521	9.635

1. Photobioreactor in a gas chamber at 3% CO_2_. 2. Photobioreactor at atmospheric (0.04%) CO_2_ content. Measurement of pH on day 0, day 1, and day 2 (pH_0_, pH_1_, pH_2_).
